# Dermal stiffness governs the topography of the epidermis and the underlying basement membrane in young and old human skin

**DOI:** 10.1111/acel.14096

**Published:** 2024-03-12

**Authors:** Eva Roig‐Rosello, Guila Dayan, Simone Bovio, Patricia Manissier, Elisabeth Errazuriz, Patricia Rousselle

**Affiliations:** ^1^ Laboratoire de Biologie Tissulaire et Ingénierie Thérapeutique CNRS UMR 5305, Université de Lyon Lyon France; ^2^ Native Laboratoire Bezons France; ^3^ RDP Université de Lyon, ENS de Lyon, UCBL1, INRAE, CNRS Lyon France; ^4^ PLATIM‐LyMIC Université de Lyon, ENS de Lyon, Inserm, CNRS Lyon France; ^5^ Ciqle, Faculté de Médecine Lyon‐Est Lyon France

**Keywords:** cell‐matrix junctions, cellular mechanotransduction, extracellular matrix, Kruppel‐like factors, skin aging, ultrastructure

## Abstract

The epidermis is a stratified epithelium that forms the outer layer of the skin. It is composed primarily of keratinocytes and is constantly renewed by the proliferation of stem cells and their progeny that undergo terminal differentiation as they leave the basal layer and migrate to the skin surface. Basal keratinocytes rest on a basement membrane composed of an extracellular matrix that controls their fate via integrin‐mediated focal adhesions and hemidesmosomes which are critical elements of the epidermal barrier and promote its regenerative capabilities. The distribution of basal cells with optimal activity provides the basement membrane with its characteristic undulating shape; this configuration disappears with age, leading to epidermal weakness. In this study, we present an in‐depth imaging analysis of basal keratinocyte anchorage in samples of human skin from participants across the age spectrum. Our findings reveal that skin aging is associated with the depletion of hemidesmosomes that provide crucial support for stem cell maintenance; their depletion correlates with the loss of the characteristic basement membrane structure. Atomic force microscopy studies of skin and in vitro experiments revealed that the increase in tissue stiffness observed with aging triggers mechanical signals that alter the basement membrane structure and reduce the extent of basal keratinocyte anchorage, forcing them to differentiate. Genomic analysis revealed that epidermal aging was associated with mechanical induction of the transcription factor Krüppel‐like factor 4. The altered mechanical properties of tissue being a new hallmark of aging, our work opens new avenues for the development of skin rejuvenation strategies.

## Graphical abstract

The skin fulfills vital functions for humans. Its protective barrier function is provided by the epidermis, which is constantly renewed thanks to the presence of progenitor cells that are firmly attached to the underlying basement membrane and whose proliferation and differentiation give it a typical undulating topography. Our study shows that the stiffness of the dermis, which increases with aging, strongly modifies the adhesion mechanisms of epidermal cells and influences their fate by driving them into the differentiation pathway.

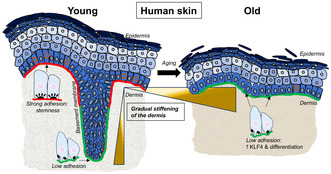

## 1 INTRODUCTION

Basement membranes (BMs) are highly organized and well‐defined planar extracellular matrix (ECM) networks located at the epithelial‐stromal interfaces of tissues. They provide a structural support to cells and regulate their behavior (Yurchenco, 2011). BMs consist of a network of self‐assembled laminins (LMs) and a network of cross‐linked collagen IV linked by the glycoprotein nidogen and the heparan sulfate (HS) proteoglycan perlecan (Breitkreutz et al., 2013). BMs can serve as signaling platforms by sequestering growth factors thereby facilitating spatiotemporal regulation of receptor‐ligand interactions (Jayadev & Sherwood, 2017). The structural organization and hydration state of ECM can have a direct impact on their mechanical properties and their capacity to generate mechanical signals to neighboring cells via a process that evolves during aging (Candiello et al., 2010; Pastor‐Pareja & Xu, 2011).

In skin, the BM that connects the outer epidermis to the underlying dermis provides essential structural support for basal keratinocytes (Rousselle et al., 2022). Several adhesion structures form at this interface, including hemidesmosomes (HDs) that connect the BM to the intermediate filament of the cell cytoplasm via the integrin, α6β4. Other structures at this interface include focal adhesions and epithelial podosomes, including β1‐integrins that are connected to the actin cytoskeleton (Michopoulou et al., 2020; Watt, 2002). These adhesion structures are components of the dermal‐epidermal junction (DEJ) and represent a specific informative niche that mediates signaling and epidermal renewal via the proliferation of stem cells and the differentiation of their progeny (Koster & Roop, 2007). In addition to the archetypal BM molecular network, the DEJ contains anchoring complexes with components that, when assembled, serve to increase the resistance of the epithelium to applied forces. These anchoring complexes connect basal keratinocytes, span the BM, and anchor the papillary dermis via the orderly assembly of specific factors including α6β4‐integrin, the LM isoform 332 (LM‐332), and collagens VII and XVII (Has & Nyström, 2015; Te Molder et al., 2021).

The DEJ in human skin exhibit a characteristic microarchitecture with an undulating pattern generated by epidermal rete ridges, which are outgrowths of the epidermis that protrude into the dermis (Lawlor & Kaur, 2015). Rete ridges surround the dermal papillae, strengthen the connection between the dermis and epidermis, and improve the mechanical properties of the skin (Langton et al., 2017). Their lengths vary according to their anatomical location; this property appears to correlate positively with the force of the mechanical stresses to which a given tissue is subjected (Topczewska et al., 2019). Interestingly, the development of bioinspired skin‐like scaffolds with microstructured waves that mimic the configuration of DEJ was found to promote the differentiation and stratification of reconstructed epithelia (Bush & Pins, 2012). When applied in vivo, these scaffolds can improve the re‐epithelialization of wounds (Malara et al., 2020). These findings suggest that these topographically‐patterned environments can communicate to resident cells and instruct them as to how they might respond to mechanical stress. At this time, the preferred position of human interfollicular epidermal stem cells (IFE‐SCs) along the DEJ remains controversial. While several groups have identified IFE‐SCs at the bases of the rete ridges, others have found them above the tip of the dermal papillae (Jones et al., 1995; Webb et al., 2004; Yamada et al., 2018). This discrepancy may be due to the heterogeneity of the cell population in the basal layer of the epidermis, as recently shown in a single‐cell RNA sequencing study that targeted interfollicular cells of the human neonatal epidermis (Wang, Drummond, et al., 2020). Scaffolds mimicking the undulated geography of the DEJ promoted in vitro epidermization (Fu et al., 2014) and revealed the accumulation of bright‐staining β1‐integrin‐positive keratinocytes at the tips of their domed structures via a mechanoresponsive mechanism. These findings support the hypothesis that stem keratinocytes localize at the uppermost edges of the rete ridges (Viswanathan et al., 2016).

As in all BMs, the DEJ thickens with aging due to alterations in its molecular composition and defects in its assembly (Khalilgharibi & Mao, 2021). The age‐associated structural and biomechanical changes resulting from crosslinks in ECM networks induced by advanced glycation end products lead to increased stiffness of the BM and its underlying dermis (Candiello et al., 2010; Ewald, 2020). Aging skin frequently exhibits DEJ flattening due to the progressive loss of rete ridges and dermal papillae (Neerken et al., 2004; Newton et al., 2015). This altered topology leads to reduced contact between the epidermis and dermis which impairs two‐way exchange and weakens epidermal anchorage and resistance to mechanical shear forces (Farage et al., 2009). Because the characteristic configuration of the DEJ may serve as critical support for epidermal homeostasis, our goal was to determine the contributions of its molecular composition to the formation and maintenance of rete ridges. We analyzed the expression of DEJ components along the rete ridges in human skin, tracked their fate, and performed experiments to determine the impact of tissue stiffness on their changes associated with aging.

## 2 RESULTS

### 2.1 Two distinct but overlapping molecular expression patterns identified along the rete ridges of the DEJ

**FIGURE 1:**
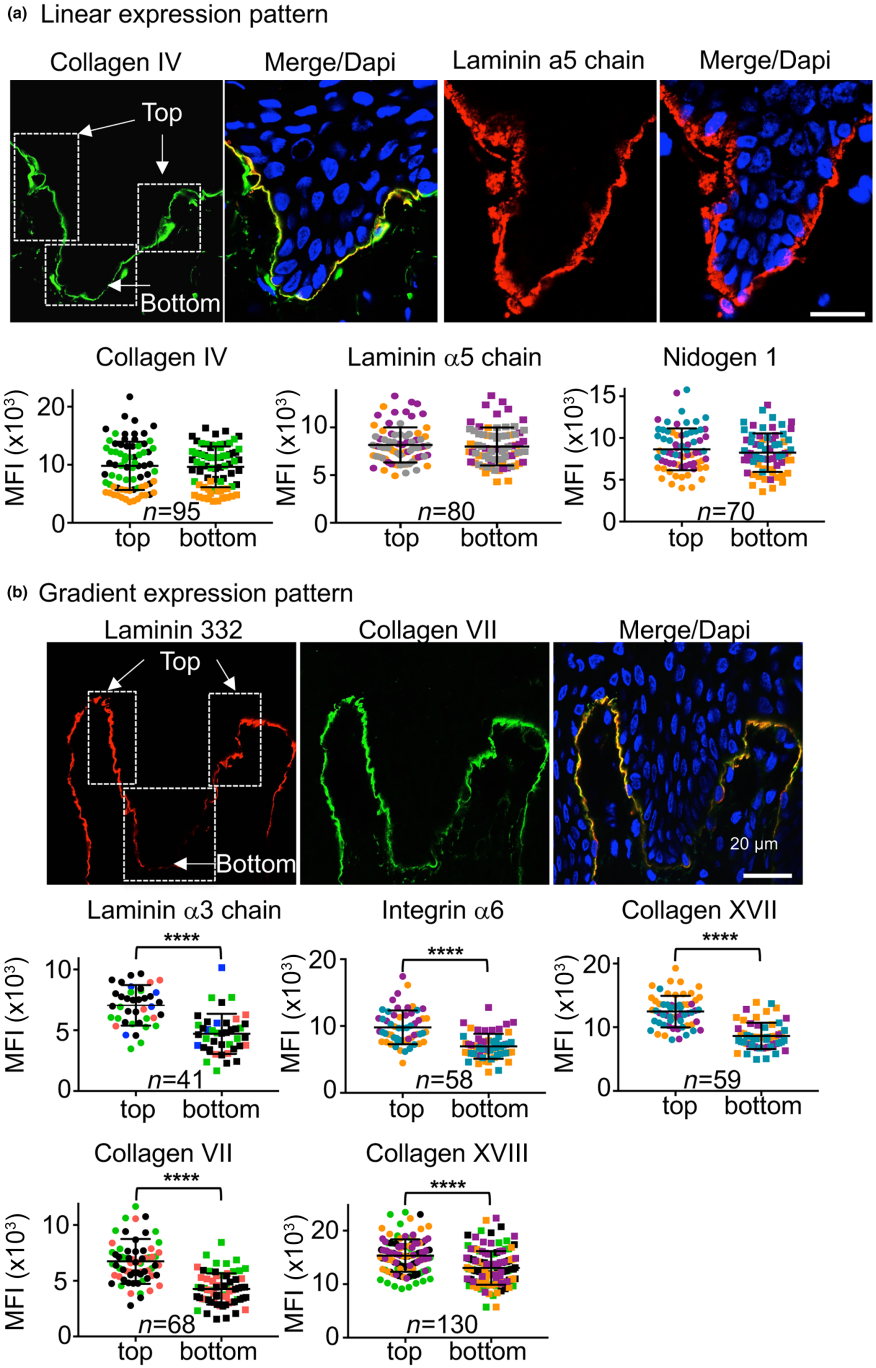
Asymmetric expression patterns exhibited by anchoring complex components along epidermal rete ridges. Immunofluorescence staining of (a) collagen IV, the LM α5 chain, and nidogen 1 and (b) the LM α3 chain, the α6 integrin, collagen XVII, collagen VII, and collagen XVIII detected in frozen sections of human foreskin. In (a), each primary antibody was detected with a FITC‐ (green) or Cy3 (red)‐labeled antibody; cell nuclei were stained with DAPI. Shown are individual and merged images of FITC‐ and/or Cy3‐labeled staining of collagen IV and LM‐511 and of LM‐332 and collagen VII. The MFIs calculated for the individual DEJ components are inserted into the image at the top and bottom of the rete ridges. Each point cloud of a particular color represents the population of rete ridges in three independent experiments that were analyzed using the color‐assigned biopsy strategy, as shown in Figure S1d, in three independent experiments with “*n*” indicated in each graph. Biopsy specimens from three different donors were analyzed in each case. Shown are mean ± standard deviations (SD) with ****p < 0.0001, determined by Student's *t* test based on the MFI detected at the top and the bottom of the rete ridge.

To identify a possible causal relationship between specific molecular constituents and the undulating pattern characteristic of the DEJ, we began by examining their expression in a cohort of human foreskin samples and targeted the expression patterns of several ubiquitous BM proteins, including collagen IV, LM‐511 (an α5, β1, and γ1 heterotrimer), and nidogen 1 (Figure 1a; Figure S1a). As expected, immunostaining revealed a continuous and homogeneous fluorescence pattern along the DEJ (Figure 1a, Figure S1a). Mean fluorescence intensities (MFIs) determined at the top and the bottom of the rete ridges as explained in Figure S1b are equivalent throughout (Figure 1a; arrows). An evaluation of the expression pattern of collagen IV with another antibody resulted in similar findings (Figure S1c). The rete ridges analyzed in this study ranged in length from 61 to 90 μm (Figure S1d). We then analyzed the expression patterns of the components of the DEJ that form anchoring complexes (Figure 1b; Figure S1e). We first evaluated the expression pattern exhibited by LM‐332 (a protein formed by the assembly of the α3, β3, and γ2 subunits) that is a core constituent of the anchoring complexes as the α3 subunit contains the major adhesion ligand for basal keratinocytes and initiates the formation of HDs (Baker et al., 1996; Carter et al., 1990; Rousselle & Aumailley, 1994). The LM‐332 aggregates are connected to a network of collagen VII‐containing fibrils; these fibrils extend into the upper regions of the papillary dermis, loop back, and are ultimately reinserted into the DEJ (Rousselle et al., 1997). These structures are reinforced by the actions of transmembrane collagen XVII, which binds to LM‐332 extracellularly and the integrin α6β4 intracellularly, and thus provides connections to the cytoskeletal elements of the HDs including plectin and BPAG1e (or dystonin) (Nishie et al., 2011). Our detailed analysis of the expression of the LM α3‐chain revealed its continuous expression along the DEJ with a gradual decrease in intensity at the base of the rete ridges (Figure 1b). The MFI of LM α3‐chain expression at the lower edges of rete ridges was significantly lower than that detected at their upper sides. This result was confirmed in experiments using two different anti‐LM‐332 antibodies (Figure S1f). Results from experiments designed to detect immunoreactive integrin α6β4, collagen XVII, and collagen VII revealed expression patterns that were identical to that of LM‐332 (Figure 1b; Figure S1e,f). The expression of collagen XVIII, known as a BM‐associated collagen (Saarela et al., 1998), was similar to that of LM‐332 as well (Figure 1b). The double immunolabeling of LM‐332 and collagen IV on the same skin section clearly shows the differences in the expression pattern of these proteins along the DEJ (Figure S1g).

Overall, the results of these experiments revealed that some (but not all) components are expressed unevenly within the DEJ. Our findings also revealed two distinct, albeit overlapping molecular networks with different expression patterns. Specifically, we found that components of the anchoring complexes exhibit variations along the length of the DEJ.

**FIGURE 2:**
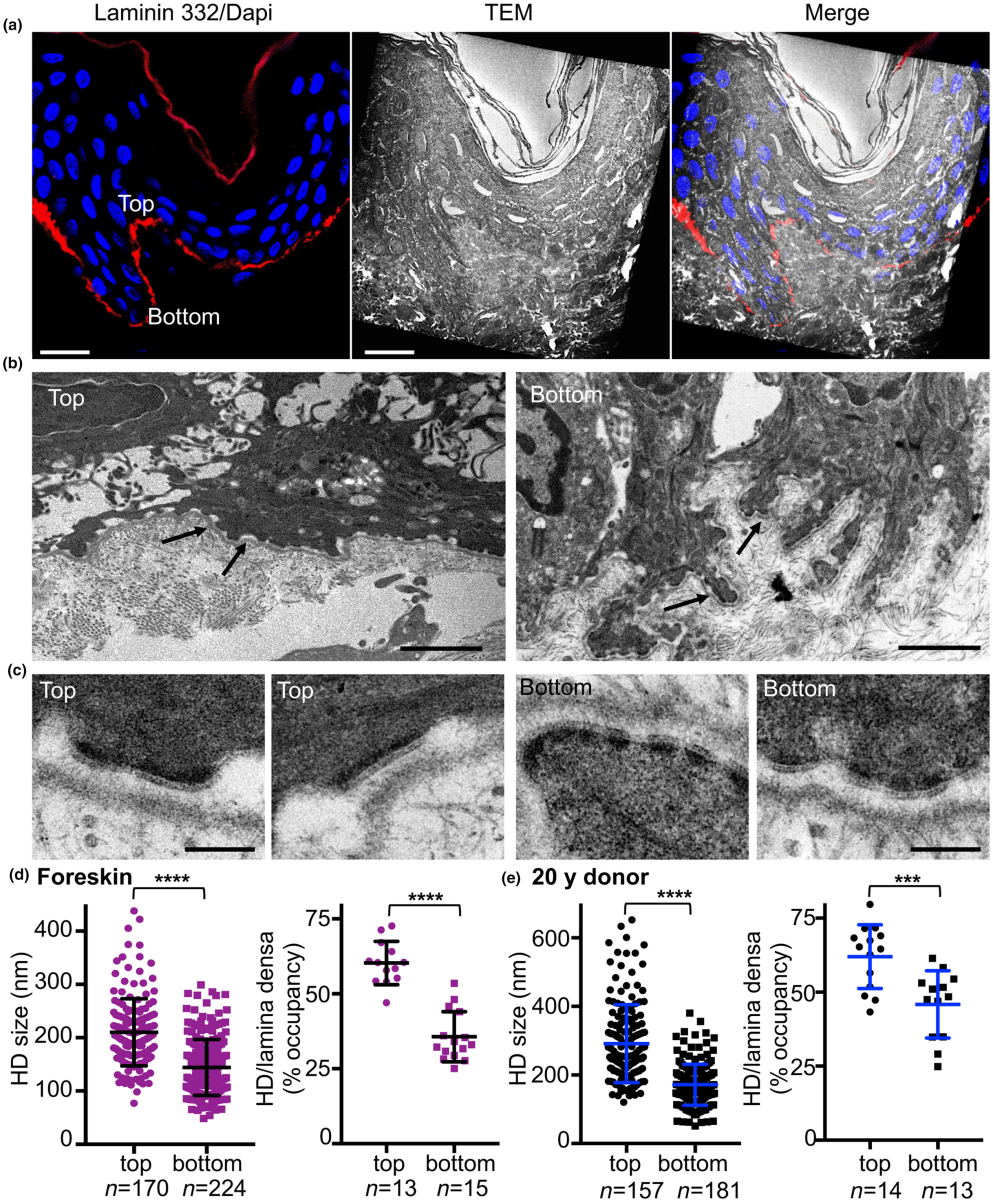
Confocal and TEM overlay microscopic analysis of the DEJ. (a) Immunofluorescence (red) depicting LM‐332 expression in the BM was projected onto the corresponding TEM photomicrograph to demonstrate the overlap of decreasing staining from the top to the bottom of a rete ridge in a foreskin biopsy specimen. (b, c) Higher magnification of the TEM image of the BM (b, at arrows) and HDs (c) at the top and bottom of the rete ridge. Scale bars indicate (a) 20 μm; (b) 2 μm, and (c) 200 nm. (d, e) Length of HD base at the top and bottom of the rete ridges and HD occupancy along the lamina densa observed in biopsy specimens of (d) foreskin and (e) the abdomen of a 20‐year‐old donor, as indicated. The number of HDs measured in each scatter plot and the number of fields used to measure HD occupancy are as shown; ***p < 0.001, ****p < 0.0001 versus control determined by Student's *t* tests.

To evaluate the potential functional impact of the aforementioned differential expression patterns, we performed an ultrastructural analysis of DEJ using transmission electron microscopy (TEM). We began with a correlative confocal and electron microscopic examination (Figure 2). Skin sections were processed for immunostaining with anti‐LM‐332 antibodies and regions with gradual changes in expression along a rete ridge were selected for processing for TEM (Figure 2a). Our review of electron micrographs that focused on large areas at the tops and bottoms of the rete ridges revealed critical ultrastructural differences (Figure 2b). The DEJ at the upper edges of the rete ridges and over the dermal papillae appeared quite flat and linear; this permitted the keratinocytes to stretch and expose their basal side to a flat surface (Figure 2b, left panel). By contrast, we observed dramatically different features at the interface between keratinocytes and DEJ at the lower edges of the rete ridges. At these sites, we observed finger‐like cytoplasmic microprojections that extended from the cell surface that penetrated the superficial dermis (Figure 2b, right panel). This notched organization resulted in a considerable increase in the contact area of the cell membrane with the DEJ. At the lower edges, each basal keratinocyte occupied an average of 21 μm, compared to the average of 15 μm on the upper surface (Figure S2a). Our review of several higher‐magnification images provided us with a more detailed analysis of the HDs in these two regions (Figure 2c). Of note, HDs appear as electron‐dense thickenings of the ventral plasma membrane into which mechanically‐resilient keratin intermediate cytoskeletal filaments are inserted, thereby firmly attaching the cells to the BM (Te Molder et al., 2021). To gain insight into the potential ultrastructural differences exhibited by HDs, we evaluated their length and the extent to which they occupied space along the lamina densa (Figure 2c,d). While the length of the HDs at the top of the ridges ranged from 77 to 438 nm (mean, 210 ± 4.83 nm), the HDs at the bottoms of the ridges were much smaller, with lengths that varied from 54 to 298 nm (mean, 144 ± 3.5 nm; Figure 2d). Furthermore, the HDs occupied an average of 60% of the length of the lamina densa at the upper ridges compared to 35% in the lower sections (Figure 2d). This experiment was repeated with a sample of abdominal skin from a 20‐year‐old donor (Figure 2e). In this biopsy specimen, the length of HDs ranged from 146 to 653 nm (mean, 291 ± 9 nm) at the upper edge and 51–380 nm (mean, 172 ± 4.8 nm) at the lower edge. Similar to our results in the first experiment, the extent of HD occupancy along the lamina densa decreased from 62% at the upper edge to 45% at the lower edge. Because the number of HDs/μm in regions of the cell membrane that were in contact with the DEJ did not vary significantly between the upper and lower edges of the rete ridge (Figure S2b), we attributed the observed differences in occupancy to specific differences in HD size. Overall, these results revealed that the decrease in expression of LM‐332 and other components of the anchoring complex at the base of the epidermal rete ridge correlates with a decrease in HD size.

### 2.2 Differences in the expression patterns of DEJ components diminish with age‐associated shortening of the rete ridges

**FIGURE 3:**
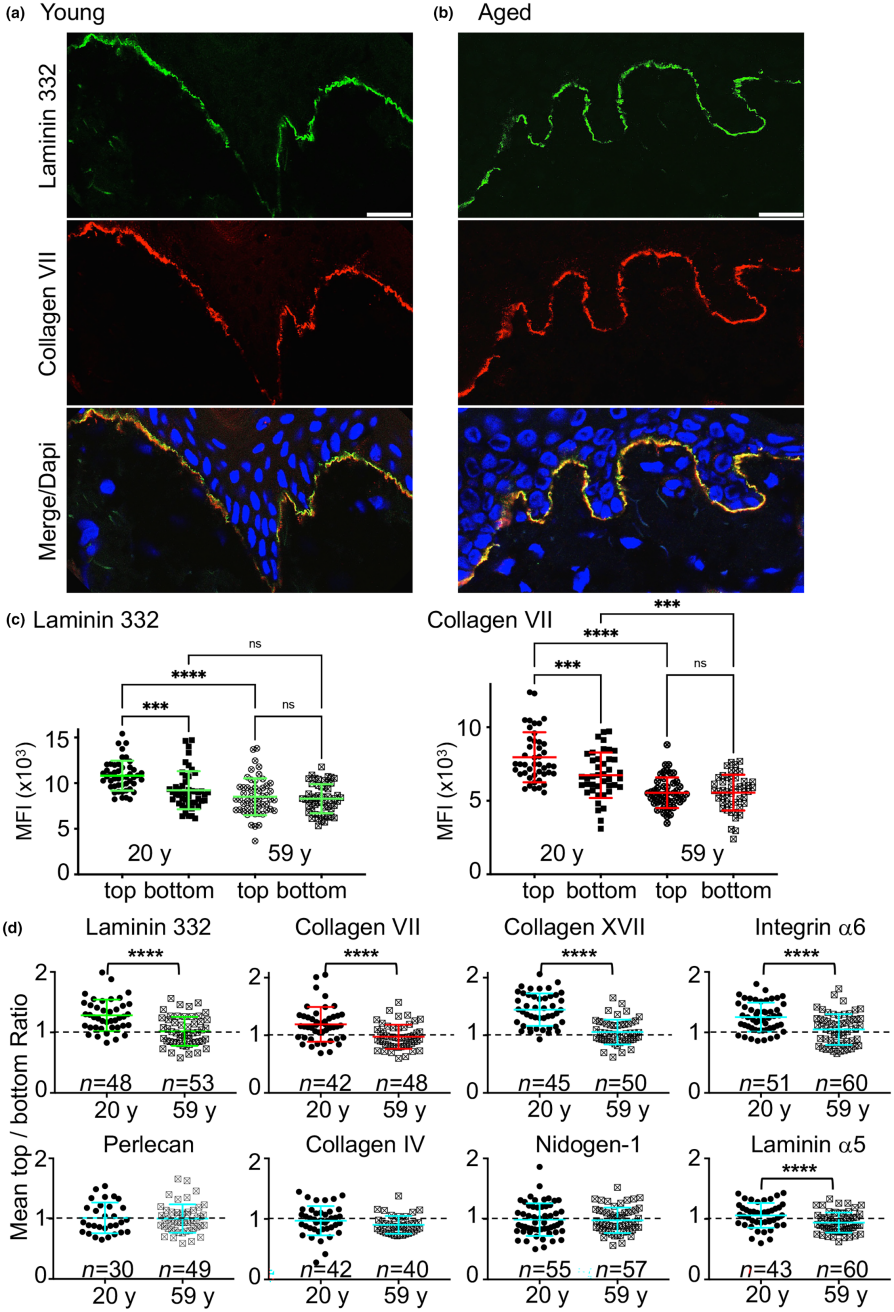
Expression of DEJ components along the epidermal rete ridges during skin aging. (a, b) Immunofluorescence staining of LM α3 chain and collagen VII on frozen sections of human abdominal skin from female donors who were (a) 20 or (b) 59 years of age. Anti‐LM‐332 pAb and the anti‐collagen VII mAb NP32 were detected with FITC‐ and Cy3‐labeled antibodies, respectively, and nuclei were stained with DAPI. Shown are individual and composite images with FITC (green)‐ and/or Cy3 (red)‐labeled staining documenting the expression of LM‐332 and collagen VII, respectively. Scale bar, 20 μm. (c) Shown are the MFIs of LM‐332 and collagen VII at the top and bottom of the rete ridges. Each point cloud represents the population of rete ridges analyzed in the abdominal skin biopsies from a 20‐ and a 59‐year‐old donor in three independent experiments. Shown are mean ± SD with ***p < 0.001, ****p < 0.0001, determined by ANOVA. (d) Comparison of the top/bottom ratios of rete ridge fluorescence for each of DEJ components evaluated in skin samples from 20‐ and 59‐year‐old donors; *n* is indicated in each graph. Shown are mean ± SD; ****p < 0.0001, *p < 0.05 was determined using Student's *t* test; ns, not significant.

Once we established the clear differential expression patterns of DEJ components, we explored these findings in a cohort of abdominal skin samples from female donors between the ages of 20 and 59 years. Immunostaining and confocal microscopy targeting the aforementioned DEJ components revealed that their expression patterns in the abdominal skin sections from the youngest female donors (i.e., those aged 20 years) were identical to those shown in the foreskin samples (Figure S3a,b). Likewise, the previously‐tested ubiquitous BM components (to which perlecan was added) were distributed homogeneously along BM (Figure S3a). Similar to the foreskin samples, the components of the anchoring complex also consistently exhibited expression patterns with decreasing intensity going from the top to the bottom on the rete ridges (Figure 3a; Figure S3b). This gradient could not be detected in skin samples from the oldest donor; as shown in Figure 3c; Figure S3b, the top and the bottom of the rete ridges in this sample revealed expression patterns of the anchoring complex proteins of equivalent intensity.

**FIGURE 4:**
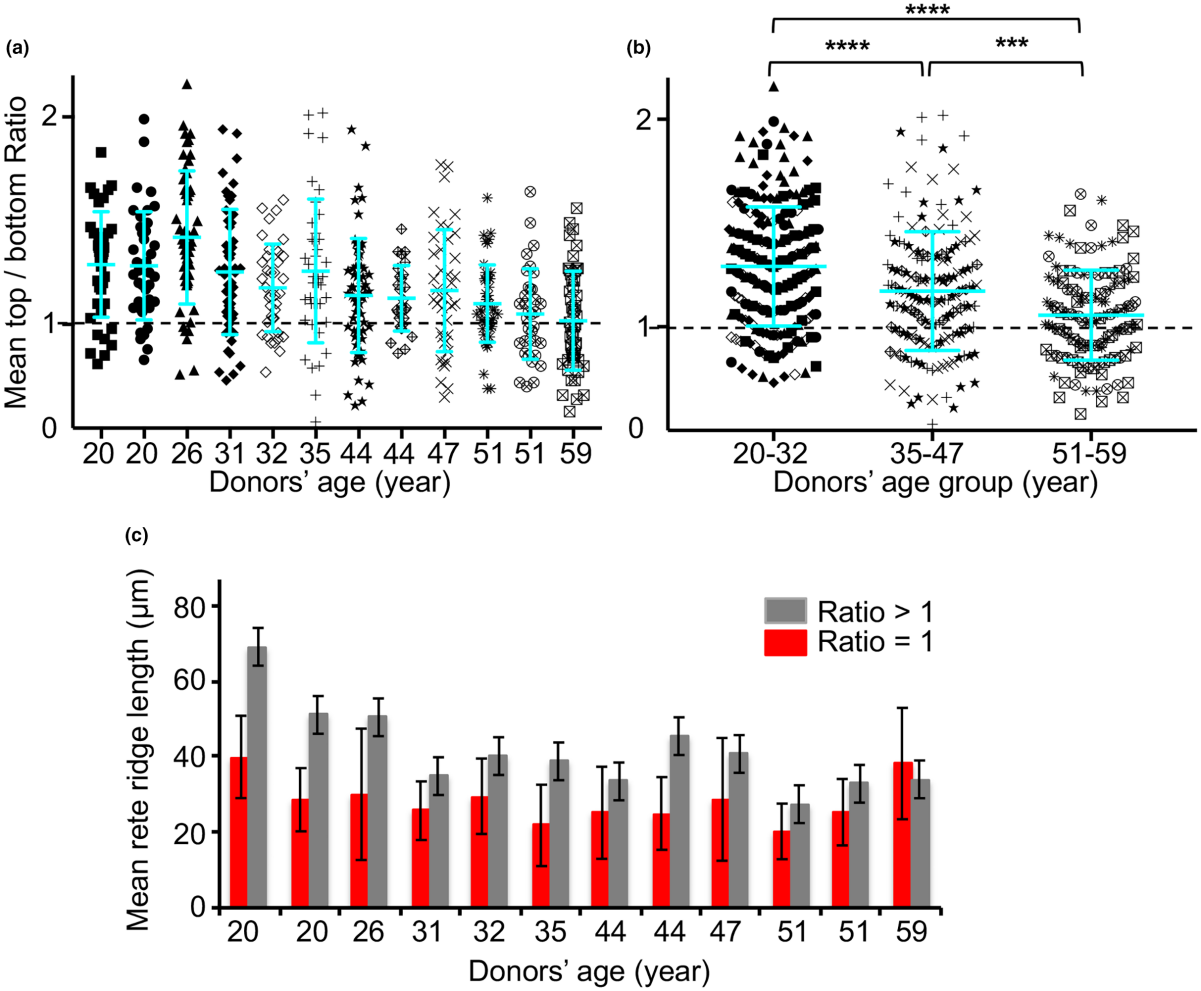
The expression pattern of LM‐332 in the DEJ changes with age. (a) Determination of the ratios LM‐332 expression at the top versus the bottom of the rete ridge in the DEJ of skin samples from donors of increasing age. All ratios were determined from the results of three independent experiments and are presented as mean ± SD. (b) Values for the top/bottom ratios are divided into three non‐overlapping age groups as shown. Results are presented as mean ± SD; ***p < 0.001, ****p < 0.0001 was determined by ANOVA. (c) Lengths of the epidermal rete ridges that have developed or lost a top/bottom LM‐332 gradient pattern.

Although the fluorescence intensity of all DEJ component tested decreased overall in the skin samples from older subjects (Figure 3a; Figure S3c–e), the expression level of anchoring complex components detected at the top of the rete ridges dropped dramatically and approached or sometimes even reached the level exhibited at the bottom (Figure 3c). This indicates the greater loss of immunoreactive protein at the top of the rete ridges. Direct LM‐332 immunostaining with a Cy3‐labeled antibody yielded similar results; a possible technological bias was thus excluded (Figure S3c). Measurements were performed for each component of DEJ (Figure S3d) that permitted us to present these findings as ratios of immunoreactive protein expressed as the top versus the bottom of the rete ridges in both young and aged skin (Figure 3d). As shown in Table 1, the top/bottom ratios of these components were >1 in samples from younger donors and gradually decreased to one during aging; by contrast, the ratios corresponding to ubiquitous BM components remained stable throughout despite decreasing levels of expression (Figure 3d; Figure S3d,e). The top/bottom ratio for LM‐332 expression in a cohort of donor skin samples decreased progressively with age; equal staining intensity of staining was observed at these two sites in biopsy specimens from the oldest donors (Figure 4a). The donor cohort was divided into three age groups; we observed a significant decline in the top/bottom ratio progressing from the youngest to the oldest cohort, thereby demonstrating the loss of LM‐332 gradient expression (Figure 4b). The lengths of the rete ridges also decreased gradually with age, ranging from an average of 62 to 36 μm in skin samples from a 20‐year‐old donor and a 59‐year‐old donor, respectively (Figure S4a). To determine whether the loss of gradient coincided with the shortening of the rete ridges, we assigned their top/bottom ratio to their length (Figure 4c). Our findings revealed that, regardless of the source of the biopsy sample, the rete ridges with diminished or absent gradients were universally shorter than those in which the gradient was maintained. Very few of the rete ridges in young donors were devoid of an LM‐322 gradient; the number of gradient‐free ridges increased gradually, ultimately reaching 50% in the skin of older donors (Figure S4b,c).

**TABLE 1:** Mean top/bottom ratio of DEJ components expression in a 20‐year‐old and a 59‐year‐old biopsy.

DEJ component	Ratio value		SEM	*p*‐Value
	Young	Aged		
Laminin 332	1.28	1.01	0.05	<0.0001
Collagen VII	1.19	0.99	0.05	<0.0001
Collagen XVII	1.45	1.06	0.05	<0.0001
Integrin α6β4	1.25	1.04	0.047	=0.0004
Collagen IV	1.00	0.90	0.44	>0.5 (NS)
Laminin 511	1.04	0.96	0.035	0.0382
Nidogen	0.99	0.98	0.045	>0.5 (NS)
Perlecan	1.01	1.00	0.056	>0.5 (NS)
Collagen XVIII	1.34	1.25	0.048	>0.5 (NS)

*Note*: *p*‐Values were determined using Student's *t* test; NS, not significant.

Collectively, our results revealed that the DEJ undergoes molecular changes during skin aging, including the gradual loss of the gradient of the anchoring complexes along the rete ridges.

**FIGURE 5:**
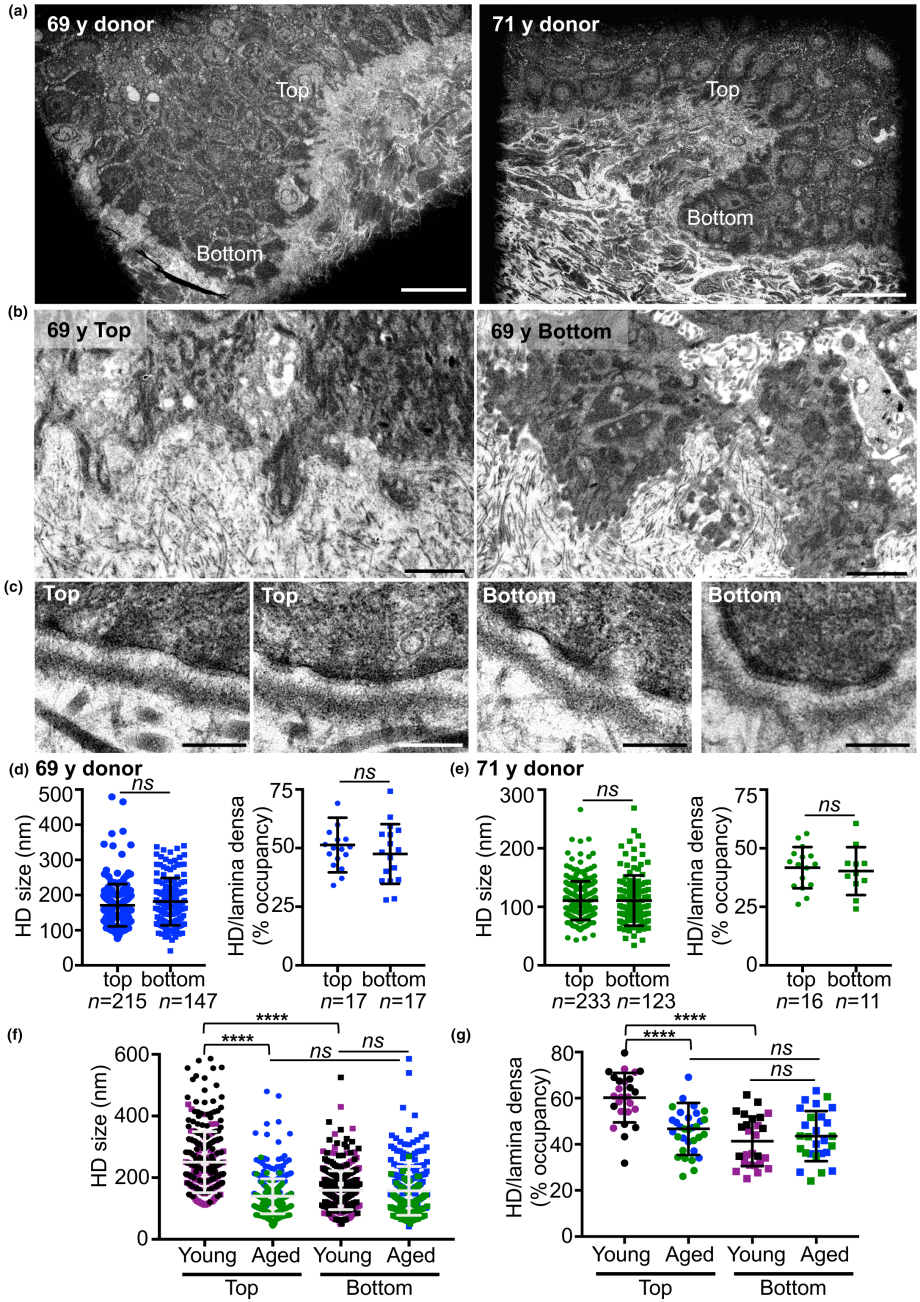
TEM analysis of the DEJ in aged skin and changes experienced over its lifespan. (a) TEM microscopic images of rete ridges in the skin of a 69‐year‐old and a 71‐year‐old female donor. (b, c) Higher magnification TEM image of the (b) BM and (c) HDs at the top and the bottom of the rete ridge. Scale bars, (a) 20 μm, (b) 2 μm, and (c) 200 nm. (d, e) Length of HD bases at the top and bottom of the rete ridges and HD occupancy in skin samples from a (d) 69‐year‐old and (e) 71‐year‐old female donor. Also shown are the number of HDs measured in each scatter plot and the number of fields used to measure the extent of HD occupancy. (f, g) Comparison of the size of HDs (f) and the extent of HD occupancy (g) at the upper and bottom of the rete ridges in younger and older donor skin. Significance was determined using a one‐sided ANOVA with Tukey's post‐test. Mean ± SD are reported as ****p < 0.0001; ns, not significant.

To explore the ultrastructural consequences of these findings, we performed TEM on skin biopsies collected from donors who were 69 and 71 years of age (Figure 5a). We noted attenuation of the finger‐like cytoplasmic microprojections of the lower cells that resulted in rounded cells with smoother edges (Figure 5b; Figure S5a). By contrast, the cell/DEJ boundaries at the upper edges appeared slightly more tortuous. Other age‐related DEJ defects were apparent, including the detachment of keratinocytes and doubling of the lamina densa at the upper edges of rete ridges (Figure S5b, at arrows) as well as incomplete nascent HDs (Figure S5b, at asterisks).

Measurements of the lengths of basal keratinocyte contact with the DEJ in specimens from these older donors revealed an average length of 23 μm above and 16 μm below the rete ridges (Figure S5d); this result is the reverse of that found in biopsy specimens from young donors (Figure S2a). Similarly, the HDs evaluated in the skin biopsy specimen from a 69‐year‐old donor were an average of 171 ± 4.1 nm at the top and 181 ± 5.5 nm at the bottom of the rete ridges, with no statistically significant differences (Figure 5c,d). Likewise, HD occupancy remained at ~50% at the top and the bottom of the rete ridges (Figure 5d). Similar results were obtained from an analysis of these parameters in a biopsy specimen from a 71‐year‐old donor. Our findings from this second specimen included HDs of ~110 nm and an HD/lamina densa ratio of ~41% at both sides (Figure 5e; Figure S5c). The number of HD/μm at both the top and bottom of the rete ridges remained the same in both older specimens examined (Figure S5e).

Examination of HDs revealed that aging is accompanied by a drastic reduction in the size of HDs on the upper side of the epidermal ridges (from 250 to 140 nm). By contrast, the size of HDs on the lower side did not change (average size, 158 nm) (Figure 5f). The incidence/occupancy of HDs on the BM on the upper side of the rete ridges ranged from 60% in the younger donors to 46% in the older donors. By contrast, this percentage remains constant on the lower side of the ridges (average 42%) regardless of age (Figure 5g). Overall, these data indicate that changes of the expression of the DEJ components that is observed with aging corresponds to the gradual disappearance of the rete ridges. They also reveal that the size of the HDs on the upper side gradually decreased to match those on the lower side.

### 2.3 Dermal matrix stiffness restricts basal keratinocyte adhesion mechanisms

Because previous studies suggested that external mechanical stresses can influence rete ridge morphogenesis by activating keratinocyte proliferation (Topczewska et al., 2019; Wu et al., 2013), we performed a series of experiments designed to address the role played by the surrounding ECM. To address this question, we analyzed the stiffness of the ECM surrounding the rete ridges by atomic force microscopy (AFM). For this purpose, unfixed sections were prepared from frozen tissue blocks and used in experiments that examined force‐volume AFM measurements, as previously described for the mechanical characterization of the ECM (Calò et al., 2020).

**FIGURE 6:**
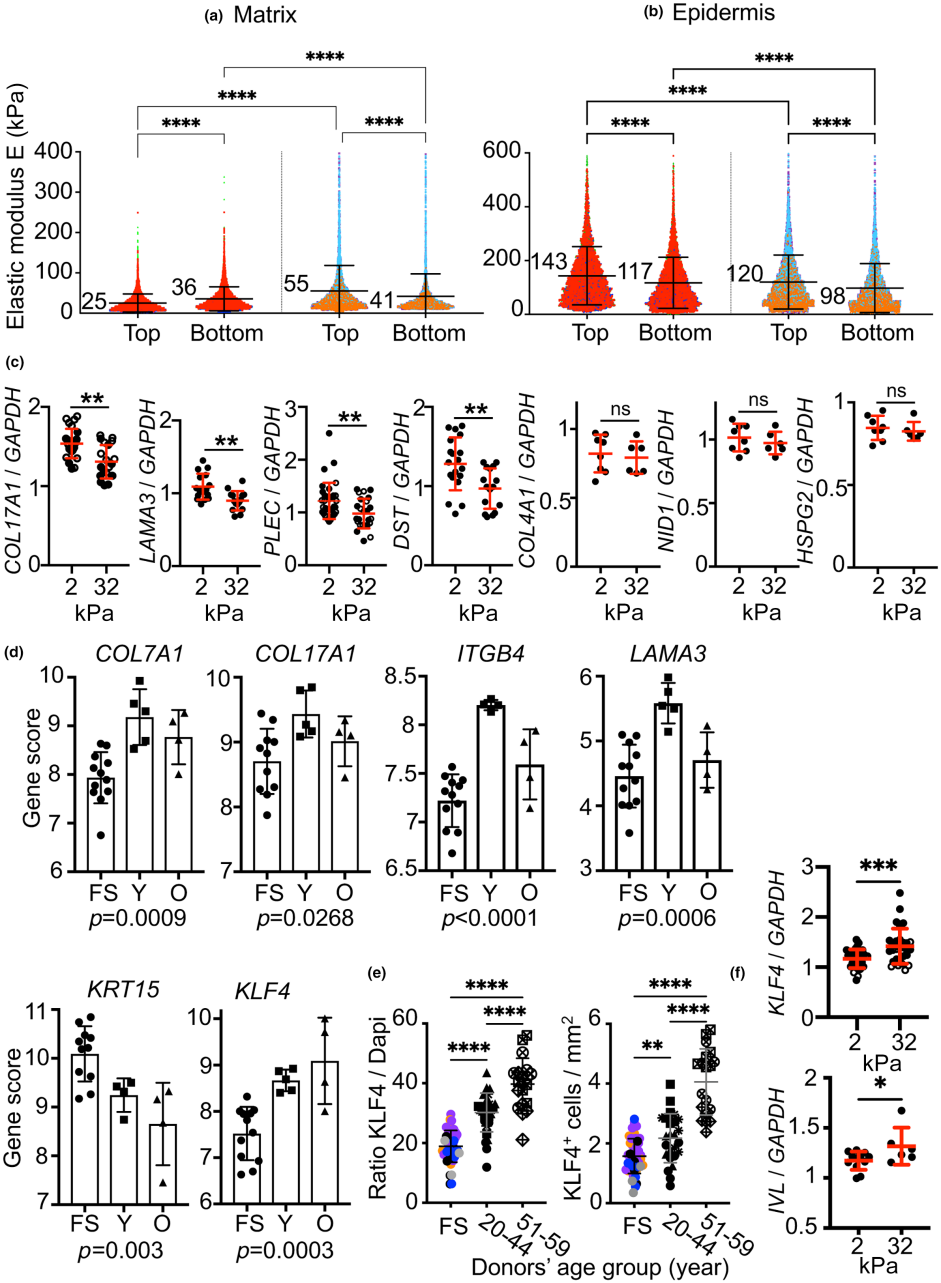
Extracellular matrix stiffness determines the fate of the HDs. (a, b) AFM assessment of human skin. Elastic modulus (*E*) values of the dermal ECM and epidermis at the top and bottom of rete ridges of abdominal skin biopsies from young and old donors. Each scatter plot represents the *E* of the force curve from 1 of the 256 spots in 10 rete ridges of each skin biopsy specimen from six independent donors, including (a) three younger donors at 20 (red), 20 (blue), and 26 (green) years of age, and (b) three elderly donors at 51 (orange), 51 (blue), and 59 (purple) years of age. Shown are mean ± SD with ****p < 0.0001; ANOVA. (c) Expression of *COL17A1, LAMA3, PLEC, DST, COL4A1, NID1 and HSPG2* mRNA in primary foreskin (strain 1, full round symbol) and abdominal skin (strain 2; from a 36‐year‐old donor, empty round symbol) keratinocytes cultured for 48 h on supports with stiffness of 2 and 32 kPa, as indicated. The graph summarizes data from three independent experiments for each of the strains evaluated. Each symbol represents the gene/*GAPDH* ratio determined for a single well in each experiment. Mean ± SD are presented, with significance at **p < 0.01 as determined by Student's *t* tests. (d) Histograms generated from the mRNA expression data reflect the differential expression of six genes in the foreskin (FS) compared to the abdominal skin of young (Y) and old (O) donors determined by NanoString nCounter® analysis. Each symbol corresponds to a single donor sample and *p*‐values determined by ANOVA are shown for all comparisons. (e) Determination of the KLF4/DAPI immunostaining ratio and number of KLF4 expressing keratinocytes in the epidermis of human foreskin and abdominal skin samples, the latter divided into two age groups as shown. Each group includes skin samples from at least three different donors (color and symbol codes included in Figures S1a and S4a). Each independent experiment was repeated twice. Mean ± SD are presented with ****p < 0.0001 and **p < 0.01 determined by ANOVA. (f) Expression of *KLF4* and *IVL* mRNA in primary keratinocytes isolated from strains 1 and 2 as indicated in (c) and cultured for 48 h on supports with stiffness of 2 and 32 kPa. The graph summarizes the data from three independent experiments for each of the strains evaluated. Each symbol represents the gene/GAPDH ratio determined for a single well in each experiment. Mean ± SD are presented with ***p < 0.001 determined by Student's *t* tests.

Measurements were performed on biopsy specimens from three young and three older donors. Bright‐field optical imaging facilitated the scanning of the location of dermal ECM areas immediately adjacent to the tops and bottoms of the rete ridges (Figure S6a) as well as the acquisition of 5 × 5 μm^2^ Force Volume (FV) maps (Figure S6b). All generated force‐indentation curves were fitted with a standard Hertz model which permitted us to generate apparent elastic (E, Young's) modulus maps (Figure S6b). The mean *E* values determined for the biopsy specimens from donors who were 20, 20, and 26 years of age were significantly lower in the ECM at the top edges of the rete ridges (25 ± 0.28 kPa) than at the bottom (35.6 ± 0.36 kPa) (Figure 6a), which indicates the presence of a stiffness gradient along the rete ridges.

As anticipated, and in agreement with previous reports, values for *E* measured in both areas of the dermal ECM were higher in specimens from older donors (those who were 51, 51, and 59 years of age). We even found that the mean *E* was greater at the upper edges of the rete ridge (55 ± 0.93 kPa) than at the lower edges (42 ± 0.86 kPa) in these specimens (Figure 6a). These data reveal that the increase in the ECM stiffness with aging is accompanied by reversal of the stiffness gradient along the rete ridges. As previously described (Feng et al., 2022), our results revealed that the epidermis exhibited a higher degree of stiffness compared to the underlying dermis layer (Figure 6b). The *E* determined for the epidermis in the lower part of the ridges (117 ± 1.18 kPa) was slightly lower than that measured in the upper sections (143 ± 1.33 kPa); both values decreased significantly in specimens from older donors.

To determine whether stiffness has an impact on the expression of anchoring complex components, we cultured primary keratinocytes on stiff surfaces and examined their expression by polymerase chain reaction (PCR; Figure 6c). Two strains of human keratinocytes cultured on a low stiffness surface (2 kPa) remained round, convex, and slightly spread, whereas those cultured on a high stiffness surface (32 kPa) were spread out and frequently exhibited a differentiated phenotype (Figure S6c). Expression of the genes involved in HD formation, including encoding collagen XVII (*COL17A1*), LM‐332 (*LAMA3*), plectin (*PLEC*), and BPAG1e (*DST*) was significantly reduced in cells plated on the rigid surface while expression of the BM genes encoding collagen IV (*COL4A1*), nidogen‐1 (*NID1*) and perlecan (*HSPG2*) remained stable (Figure 6c; Figure S6d).

To assess the impact of changes in rete ridge shape with aging on the expression of genes involved in epidermal homeostasis, we performed microdissections on skin samples across the age spectrum. We extracted RNA and performed nanostring transcriptomic analyses with a keratinocyte‐specific gene panel that included DEJ components, stem cell markers, mechanotransduction pathways, and proliferation markers to determine the changes in gene expression associated with aging (Figure S6e). Of all the genes evaluated, significant changes in gene expression were observed only among those encoding components of the anchoring complexes (Figure 6d). Specifically, we found higher levels of *COL7A1*, *COL17A1*, *ITGB4*, and *LAMA3* transcripts in the epidermis in abdominal skin samples from young donors than in the foreskin samples and decreased levels in abdominal skin samples from the older donors. As anticipated, our results revealed that the level of *KRT15* transcripts decreased with donor age, regardless of their anatomical localization in the skin. By contrast, levels of *KLF4* gene transcript increased steadily (Figure 6d). To confirm and reinforce the relevance of this latter finding, we examined the expression of immunoreactive KLF4 in the epidermis of abdominal skin samples from donors of increasing age (Figure S6f; Figure 6e). The ratio of KLF4 to DAPI staining, as well as the number of KLF4 expressing keratinocytes in the epidermis, underwent a regular and significant increase that paralleled the increasing age of the donors. We performed cell culture experiments and established a causal relationship between *KLF4* expression and the increasing stiffness of the ECM. We found a significant increase in *KLF4* transcription in keratinocytes cultured on a rigid support (Figure 6f; Figure S6g). Taken together, our results indicated that the stiffness of the ECM has a profound influence on the HD‐mediated adhesion of basal keratinocytes and promotes their proliferation and differentiation. The increase in stiffness observed in response to aging disrupts HD‐mediated adhesion and accelerates the entry of basal cells into the differentiation program as confirmed by the increase in expression of the gene coding for involucrin in our model (Figure S6g).

## 3 DISCUSSION

We have performed an in‐depth analysis of DEJ in human skin and found that the components contributing to the establishment of keratinocyte adhesion mechanisms exhibit spatially and temporally varying expression levels throughout lifetime. We determined that components constituting anchoring complexes (LM‐332, integrin α6β4, collagens XVII and VII) are differentially expressed in a top‐to‐bottom gradient along epidermal rete ridges in skin of young donors. This is consistent with a previous work (Yamada et al., 2018), which reported that LM‐332 was expressed predominantly at the tips of dermal papillae, a site where IFE‐SCs were localized. The importance of LM‐332/α6β4 integrin interaction is known as essential for maintaining epidermal stem cells via a Yes‐associated protein (YAP)‐dependent mechanism (De Rosa et al., 2019) and for controlling their entry into the differentiation program (Tayem et al., 2021; Yamada et al., 2018). Several research groups reported the consistent localization of slow‐cycling stem cells at the tip of dermal papillae (Jensen & Watt, 2006; Legg et al., 2003); our results documenting the high expression levels of anchoring complex components at this site are consistent with these findings. Slow‐cycling stem cells express high levels of integrin α6 (Webb et al., 2004) and collagen XVII has been identified as a niche component required for their appropriate function and symmetrical division (Liu et al., 2019; Matsumura et al., 2016; Natsuga et al., 2019; Wang et al., 2022; Wang, Drummond, et al., 2020; Watanabe et al., 2017). Our correlative confocal and TEM observations revealed that stepwise changes in the expression of anchoring complex components in the DEJ are accompanied by distinctive ultrastructural features. We identified a planar interaction zone with large HDs (including ~210 nm in the foreskin and ~ 291 nm in abdominal skin specimens) as a characteristic of the DEJ at the top of the dermal papillae. By contrast, the keratinocyte contact area at the bottom of the rete ridges was almost twice as long and highly irregular, as it included membrane protrusions that penetrated the dermis accompanied by smaller HDs (~144 nm in the foreskin and ~171 nm in the abdominal skin specimens). These findings suggested differences in both keratinocyte adhesion and signal transduction at these sites. They also suggest that the collagen VII/LM‐332 axis and the corresponding receptors, collagen XVII and integrin α6β4 found at the upper surface of dermal papillae might induce the formation and maturation of large HDs along a flat surface, thereby providing the firm adhesion and mechanical stability necessary for stem keratinocyte maintenance. The significant elongation of the interface between the cells and the BM at the base of rete ridges was associated with the formation of finger‐like protrusions possibly reflecting the traction generated by cells migrating to and penetrating the dermis. The reduced size of the HDs in these areas suggests that a different type of cellular interaction may be taking place that is consistent with cell movements and traction. Our observations in human tissue are consistent with those recently reported by Wang, Zuidema, et al. (2020) who found that HDs limit the capacity of keratinocytes to exert traction on their substrate. According to their finding that the α6β4 integrin binding to LM‐332 reduced keratinocyte spreading and the maturation of β1‐integrin‐mediated focal adhesions, we can hypothesize that the progressive reduction of the LM‐332/ α6β4 integrin axis at the bases of rete ridges would permit focal adhesions associated with the actin cytoskeleton to be established and mature; these cells would then be able to exert traction that would enable them to dig a path into the dermis and form a rete ridge.

One of the most prominent feature of skin aging is the shortening and gradual disappearance of the rete ridges accompanied by the decreased expression of epidermal stem cell markers (Giangreco et al., 2010) leading to the thinning of the epidermis and weakening of its regenerative capacity. Our detailed examination of DEJ components in a collection of human skin from younger to older donors revealed the progressive decline of the components of the anchoring complex at the upper edges of rete ridges to ultimately match the expression levels at the lower edges. This corresponded to the progressive disappearance of large HDs at the upper rete ridges and their replacement by small HDs, resulting in a uniformly‐reduced HD contact area in shortened rete ridges. Our results corroborate previous human and mouse studies that revealed how downregulation of collagen XVII during aging or in cases of epidermolysis bullosa led to HD instability and atrophy of the epidermis with loss of epidermal stem cells (Liu et al., 2019). Studies of bullous pathologies in humans or transgenic mice in which the expression of LM‐332, integrin α6β4, or collagen VII is reduced or absent demonstrated the essential roles of these proteins in the stability of HDs (Prodinger et al., 2019). Similarly, Tasanen et al. (2004) reported that the correct integration of LM‐332 into the ECM requires collagen XVII. Studies focused on the reconstitution of cornea revealed a role of collagen VII in the formation of HDs (Jones et al., 1998).

ECM stiffness determines cell behavior and gene expression via mechanisms involving integrin sensing and mechanotransduction (Engler et al., 2006; Janmey et al., 2020). Our measurements of *E* in the dermis and epidermis agree well with values previously obtained of human skin in vivo (Feng et al., 2022; Li et al., 2012; Zhou et al., 2021). Our results showing the marked increase in stiffness in the ECM adjacent to the DEJ of the rete ridges moving from the top (25 kPa) to the bottom (36 kPa) suggest that the increasing mechanical constraints imposed on keratinocytes along the rete ridges may influence their growth. Migration and/or growth of epithelial cells in response to stiffness has been reported previously, including directional growth of epithelial cells in response to a substrate stiffness gradient (Saez et al., 2007). The epidermis is a mechanosensitive tissue; keratinocytes actively sense the physical properties of their environment (Evans et al., 2013). They sense increased tension (Knies et al., 2006) and undertake increased mitotic activity via integrin‐mediated mechanosensing pathways (Kenny et al., 2018). Our measurements of *E* in the ECM of skin samples from older donors revealed that, as expected, stiffness had increased significantly (Haydont et al., 2019). However, the increase in stiffness was more marked at the top (55 kPa) than at the bottom of the rete ridge (41 kPa), reversing the direction of the stiffness gradient. This profound increase in *E* at the top of the rete ridge may explain well the drastic phenotypic and ultrastructural changes that we observed in the DEJ, namely, the diminished expression of components of the anchoring complex and the reduced HD‐mediated anchorage. Therefore, the flattening of DEJ during aging may occur in response to a stiffness‐induced weakening of the HD‐mediated anchorage of stem cells at the top of the rete ridges, which then take on the phenotypic features of the cells at the bottom, including a gradual reduction in their progenitor potential. Koester et al. (Koester et al., 2021) described a similar phenomenon in the stem cell niche of the hair follicle. Our results complement hypotheses based on a particle‐based model of self‐replicating cells on a deformable substrate in which different adhesion strengths of the cells were crucial factors contributing to the characteristic undulating pattern of a BM (Kobayashi et al., 2018). Our in vitro results revealed reductions in the expression of genes encoding LM‐332, collagen XVII, plectin, and BPAG1e in primary keratinocytes that were cultured on a rigid surface (32 kPa) compared to those cultured on a soft surface (2 kPa). These findings support our hypothesis that increased substrate stiffness contribute to the modulation of HD formation. Results from previous studies featuring mammary cells revealed that increased ECM stiffness stimulated β1 integrin signaling and proliferation (Schedin & Keely, 2011), and prevented normal clustering of α6β4‐integrins in HDs (Chaudhuri et al., 2014). We suggest that an optimal stiffness might be required to maintain epidermal stem cells via the formation of large HDs. The basal keratinocytes located at the different levels of the rete ridge perceive and absorb the increasing stiffness of the dermal ECM with age, which is related to the progressive increase of enzymatic and stochastic non‐enzymatic intra‐intermolecular cross‐links between molecules with slow turnover, such as fibrillar collagens and elastin (Selman & Pardo, 2021). Our genomic analysis of transcripts expressed in microdissected human skin rete ridges revealed that genes encoding components of anchoring complexes underwent significant age‐associated changes. They also revealed the progressive increase in expression of the transcription factor, Krüppel‐like factor 4 (KLF4), that we confirmed at the protein level in skin sections. KLF4 supports the epidermal barrier function by promoting terminal keratinocyte differentiation and plays a critical role in stem cell biology (Bialkowska et al., 2017). Recent research in both mice and humans revealed that activation of KLF4 can lead to inhibition of the YAP1/TAZ‐TEAD transcriptional network which has an essential function in maintaining keratinocytes in their basal, undifferentiated state (Elbediwy et al., 2016; Yuan et al., 2020). Furthermore, stable downmodulation of KLF4 promotes immaturity, self‐renewal, and the regenerative capacity of adult human keratinocyte progenitor cells (Fortunel et al., 2019). Findings from our study revealed that keratinocytes cultured in a rigid environment not only downregulate the components of the anchoring complexes, they also activate the expression of KLF4. The possibility of linking HD‐dependent adhesion to the actions of this transcription factor provides us with new insights into the mechanoregulatory mechanisms that contribute to skin aging. Our study showing an increase in KLF4 expression in the epidermis during aging suggests a detrimental effect on the mechano‐regulated maintenance of basal progenitor cells.

Overall, our results suggest that the epidermal BM takes shape in response to healthy mechanical signals from the surrounding ECM. These signals instruct and maintain epidermal homeostasis and the properties of basal progenitor keratinocytes by regulating the expression of components of the anchoring complex. The altered mechanical properties of tissue, being now considered a new hallmark of aging (Schmauck‐Medina et al., 2022), modify these signals and lead to the disruption of epidermal homeostasis as reflected in a refashioned geometry of the DEJ.

## 4 MATERIALS AND METHODS

### 4.1 Collection of skin specimens

Foreskin samples were collected from eight male infants (mean age, 4 years old) who were undergoing circumcision. Biopsies of UV‐unexposed skin were collected from female patients who had undergone abdominal reduction following ethical and safety guidelines according to French regulations. Verbal and written informed consent were obtained from all participants who provided skin samples according to declaration no. DC‐2008‐162 that was submitted to the Cell and Tissue Bank of the Hospices Civils de Lyon. The 14 female participants enrolled in this part of the study included five elderly donors (aged 51–71 years), four middle‐aged donors (ages 35–47 years), and five young donors (aged 20–32 years). Surgical specimens were embedded in Tissue‐Tek OCT (Microm Microtech, Brignais, France) and stored at −80°C or processed for paraffin embedding.

### 4.2 Correlative confocal and electron microscopy

Skin samples were fixed in 4% glutaraldehyde in PBS for 4 h at 4°C and then immersed successively in 10%, 20%, 30%, and 40% sucrose in PBS. The samples were frozen in liquid nitrogen; 20‐μm frozen sections were prepared and mounted on 10 × 10 grids with 0.1‐mm squares at five positions on correlative microscopy coverslips (CMC34A, Pyser‐SGI Limited, Kent, UK). The sections were incubated for 1 h with the pAb anti‐LM‐332 diluted in PBS with 5% NGS, washed in PBS, and incubated with Cy3‐conjugated secondary antibodies and 0.5 μM DAPI solution. Slides were mounted with ProLong Gold Antifade Mountant (Invitrogen). Fluorescence images were acquired using a confocal laser scanning microscopy (Zeiss LSM 800) and the slides were processed for TEM. The coverslips were washed in PBS and fixed with 2% glutaraldehyde (Delta Microscopie, Labège, France) in a 0.1 M cacodylate buffer, pH 7.2 for 30 min at RT. The samples were washed and post‐fixed with 1% osmium tetroxide (OsO_4_) in 0.1 M cacodylate buffer, pH 7.2, at RT followed by gradual dehydration in ethanol (30%–100%), gradual immersion in an ethanol‐epon mixture, and embedding in Epon. The embedding was done by positioning a capsule (Delta Microscopies) that spanned the relevant region of interest (ROI) on the coverslip. The cover glass resin corresponding to the capsule was polymerized at 60°C for 24 h. After polymerization, the correlative coverslip was removed from the resin block. The block with the embedded ROI was cut from the coverslip in a small trapezoid shape; the remaining ROI was cut out with a diamond knife. Ultra‐thin (~100 nm thick) sections were cut on a UC7 (Leica), mounted on 150‐mesh copper grids coated with 1:1000 polylysine, and stabilized for 1 day at RT and then treated with uranyl acetate and lead citrate. The sections were examined with a Jeol 1400JEM (Tokyo, Japan) transmission electron microscope equipped with an Orius 600 camera and a Digital Micrograph.

### 4.3 Atomic force microscopy

AFM indentation experiments were performed in PBS at RT using a Bioscope II Catalyst (Bruker Nanosurfaces, Santa Barbara, CA, USA; driven by Nanoscope v. 9.13) mounted on a DMI600 inverted optical microscope (Leica, driven by Micro‐Manager 2.0‐gamma) that was equipped with a 20x dry objective. Pre‐calibrated PFQNM‐LC‐A‐CAL cantilevers (Bruker) were used with a nominal spring constant of 0.1 N/m and a nominal tip radius of 70 nm. Deflection sensitivity was calibrated by generating a force curve on a sapphire disc in PBS. The FV mode was used to measure Young's modulus (*E*). In this mode, a series of force curves were generated along a matrix defined for the ROI of the sample. For these experiments, 16 × 16 force curves were acquired for 5 × 5 μm^2^ areas, which yielded 256 curves for each zone (Figure S6B). For all measurements, the curve parameters included a ramp size of 2 μm, a ramp rate of 1 Hz, and a trigger force of 1 nN, corresponding on average to an indentation of 300 nm; this will be small enough compared to the distance between indentation positions so that they will not be influenced by one another. A minimum of 10 rete ridges were evaluated in each biopsy specimen, including the cellular side and the ECM at the top and bottom of each ridge. Hydrated sagittal cryosections (10‐μm) were examined and the *E* of the dermis and epidermis were measured. The glass slide was positioned and blocked on a motorized XY stage and 100 μL of PBS was added to the sample. The tip was driven to the ROI using an optical microscope. Each curve was analyzed using the Nanoscope Analysis software (Bruker Nanosurfaces, v. 2.0) to determine the average E. A baseline correction algorithm was used to remove any tilt and set the average to 0 force. We fit each force‐versus‐indentation curve using the classical Hertz model:

Fδ=43E1−ν2·R·δ32

where *E* is the relative apparent Young's modulus, *ν* is the Poisson ratio of the sample (i.e., 0.5 for our samples that were incompressible), as generally assumed for biological material and specifically confirmed for skin sample (Li et al., 2012), *R* is the nominal radius of the tip and *δ* is the indentation. All the fits were performed between 10% and 90% of the trigger force. All curve fitting was performed using 10%–90% of the trigger force. In its strictest sense, the Hertz model is only applicable to elastic, homogeneous, and isotropic materials. However, *E* is generally accepted as a measure of the mechanical properties of tissues responding to small strains, which is the case for AFM‐generated indentations.

### 4.4 Microdissection and RNA extraction

Twelve human skin samples were fixed in formalin and embedded in paraffin. Tissue sections (10 μm thick) were cut for laser capture microdissection. The tissue samples were mounted on PEN Membrane Glass Slides (Applied Biosystems) and dried overnight at RT. To visualize the areas of interest, the tissue sections were deparaffinized in two baths of xylene and one bath of absolute ethanol and stained with cresyl fast violet. For each tissue sample, a second slide was stained with a conventional hematoxylin–eosin stain to assess tissue morphology. Total RNA was extracted using the RNeasy FFPE kit (Qiagen, Courtaboeuf, France). RNA yield and purity were assessed using a DS 11 spectrophotometer (Denovix Inc.).

### 4.5 Cell culture

Normal human keratinocyte (NHK) cultures were established from abdominal skin samples from participants who were 20 (strain 1) and 32 (strain 2) years of age according to previously published procedures (Michopoulou et al., 2020). The cultured cells were grown in a keratinocyte growth medium supplemented with 0.15 mM CaCl_2_ (KBM‐2 BulletKit, Lonza Biosciences, Basel, Switzerland). To initiate experiments, 2 × 10^5^ keratinocytes were cultured in CytoSoft® 6‐well plates, elastic moduli 2 and 32 kPa (Sigma Aldrich), for 48 h in keratinocyte basal medium (KBM)‐2. Wells were rinsed with sterile PBS and processed for RNA extraction. Cells were photographed using a Zeiss Axiovert 40 microscope equipped with a circular differential interference contrast and coupled to a Coolsnap Fx camera (Roper Scientific, Evry, France).

### 4.6 Statistical evaluation

Unpaired *t* tests and one‐tailed analysis of variance (ANOVA) were used to evaluate the data; *p* < 0.05 was considered statistically significant. The size of the replicate measurements is indicated in the legend of each graph; the number of experimental repetitions is indicated by *n*. Individual data values are presented systematically for cases in which *n* < 6. Statistical analyses were performed using GraphPad Prism software, version 8.4.2 (GraphPad software). All results are presented as mean ± SEM.

## AUTHOR CONTRIBUTIONS

P.R. conceived and directed the study, analyzed the data, prepared the figures, and wrote the manuscript. E.R.‐R. performed and analyzed the data from the immunofluorescence and AFM experiments. S.B. developed the experimental conditions for AFM. E.E. performed the electron microscopy studies. P.R. designed the nanostring panel. P.R. and G.D. performed cell culture, microdissection, and RNA extractions and analyses. All authors have read, revised, and agreed with the submitted version of the manuscript.

## CONFLICT OF INTEREST STATEMENT

E.R.‐R. is a PhD student employee of NATIVE and received funding from the French National Association for Research and Technology. The authors declare no competing interests.

## Supporting information


Data S1:

## Data Availability

The data supporting the findings presented in this study are available from the corresponding author upon reasonable request.
