# Supplementary material for: Dermal stiffness governs the topography of the epidermis and the underlying basement membrane in young and old human skin
Source: Aging Cell. 2024 Mar 12;23(4):e14096. doi: 10.1111/acel.14096 (PMC11019137; doi:10.1111/acel.14096)
Supplement: Supplementary file 1 — Data S1: [file ACEL-23-e14096-s001.zip › Supporting Supplemental Information.pdf]

Supporting Supplemental Information

MATERIALS AND METHODS

Immunohistochemical procedures

Six micron frozen sections were fixed in glyoxal (Sigma Aldrich, Saint-Quentin-Fallavier, France) for 30 min at 4°C and then for 30 min at RT. For staining with anti-collagen XVIII, skin sections were fixed in pre-cooled methanol/acetone (v/v) for 10 min at 4°C and rehydrated with PBS. After washing, all sections were incubated with 5% normal goat serum (NGS) (Jackson ImmunoResearch, Ozyme, Saint Quentin Yvelines, France) in PBS for 30 min. Polyclonal Abs (pAbs) against LM-332 (anti-LM-332 and L132) (Bachy et al., 2008; Decline & Rousselle, 2001), collagen VII (3959 and 1854) (Christiano et al., 1996; Lunstrum et al., 1986), nidogen 1 (Paulsson et al., 1986) and GTX114587 (GenTex, CliniSciences, Nanterre, France) and monoclonal Abs (mAbs) against LM α3 chain (BM-165) (Rousselle et al., 1991), LM α5 chain (4C7; DakoCytomation, Trappes, France), α6 integrin (GoH3; Merck Millipore, Molsheim, France), β1 integrin (P1B5, Santa Cruz Biotechnology, Le Perray en Yvelines, France), collagen VII NC1 domain (NP32) (Sakai et al., 1986), collagen IV (clone COL-94 from Sigma Aldrich and MAB-1(Eurodianostica AB, Malmö, Sweden), collagen XVII (EPR18614, Abcam, Amsterdam), KLF4 (AP2725a from Abcepta, San Diego, CA, USA). After washing in PBS, Cy3- or FITC-conjugated antibodies (Jackson, Beckman Coulter, Paris) were applied for 30 min and nuclei were stained with a 0.5 µM DAPI solution. The slides were mounted with ibidi Mounting Medium (ibidi, Cliniscience). The staining procedure used to detect perlecan was described previously (Dos Santos et al., 2016). Fluorescence was visualized by confocal laser scanning microscopy (Zeiss LSM 800). Several focal planes with a depth of 0.8 µm were recorded systematically.

Image analysis and processing

Images from a 0.8 µm focal plane were analyzed using Fiji software (RRID: SCR _002285). The Mean Fluorescence Intensity (MFI) of the segments of the DEJ located at the top and bottom of each rete ridge were measured using the Bioformats plugin for spatial calibration of the image. The length of each epidermal rete ridge was measured from top to bottom, with the DEJ boundaries used as upper limits and without including different layers of epidermis. The measured length was divided into two equal parts. Fluorescence intensity was then determined by drawing a 2 µm-wide freehand line across the DEJ to define the area to be evaluated quantitatively. The MFI/μm^2 was then calculated for both halves. Rete ridges from three random images from each experiment were analyzed; three experiments were performed for each antibody and samples from three different donors.

Transmission electron microscopy

Tissues were cut into small pieces and fixed in 2% glutaraldehyde for 2 h at 4°C. The samples were washed three times for 1 h at 4°C and then fixed in 2% OsO_4 for 1 h at 4°C. The fixed tissues were then dehydrated in a series of graded ethanol washes and transferred to propylene oxide. Tissues were then impregnated with Epon A (50%), Epon B (50%), and DMP30 (1.7%). Inclusions were obtained by polymerization at 60°C for 72 h and sections were prepared using a UC7 (Leica) ultramicrotome. Area changes were first selected on semi-thin sections (1000 nm thick) stained with methylene blue Azur II. Ultra-thin sections (~100 nm thick) were then cut, mounted on 150 copper grids coated with 1:1,000 polylysine, stabilized at RT for one day, and treated with uranyl acetate and lead citrate. The sections were

examined with a Jeol 1400JEM (Tokyo, Japan) transmission electron microscope, 100Kv, equipped with an Orius 600 camera and Digital Micrograph.

Reverse transcription-polymerase chain reaction analysis

Total RNA was extracted from NHK cells cultured in CytoSoft® 6-well plates using the RNeasy MicroKit (Qiagen) according to the manufacturer's instructions. The RNA concentration of each sample was determined using a NanoDrop 2000 spectrophotometer (Thermo Fisher Scientific). Total RNA (1 µg) was used for first-strand cDNA synthesis using M-MLV Reverse Transcriptase (Roche Diagnostics, Sigma Aldrich) in the presence of an oligo(dT)15 primer (Promega, Charbonnières-les-Bains, France). Reverse transcription was performed for 50 min at 37°C. The cDNA products were amplified using FastStart Taq DNA Polymerase (Roche Diagnostics) and the following primers: *KLF4-F*, 5'-TCTCTTCGTGCACCCACTTG-3'; *KLF4-R*, 5'-GAAGAAGGTGGGGTGAGCAT-3'; *COL17A1-F*, 5'-GCAGCAGCGGCTACATAAAC-3'; *COL17A1-R*, 5'-TCGACTCCCCTTGAGCAAAC-3'; *DST-F*, 5'-AGTCCAATTCCAGCCACCAG-3'; *DST-R*, 5'-AGAACACAGAGTATACCTAAGGG-3'; *PLEC-F*, 5'-TGGCCAGCAGATCTCTCT-3'; *PLEC-R*, 5'-GGATGGCAGCAGGTCTTTCT-3'; *LAMA3-F*, 5'-ACCCAGGCCAAGGACCTGAGG-3'; *LAMA3-R*, 5'-GTGTTGCCCGATTAACATTG-3'; GAPDH-F, 5'-ATCACTGCCACCACCCAGAAGAC-3'; and *GAPDH-R*, 5'-ATGAGGTCCACCCTGTT-3'. Reactions were performed in a Biometra T-professional standard thermal cycler (Labgene, Archamps, France). The polymerase chain reaction (PCR) amplicons were visualized by agarose gel electrophoresis and staining with SYBR Safe (1:20,000) (LifeTechnologies). The gels were scanned using

a STORM imaging system (Molecular Dynamics, Sunnyvale, CA, USA). The resulting images were analyzed using ImageQuant software (Molecular Dynamics).

Nanostring experiment

RNA (100 ng) was hybridized with a panel of selected probes (Nanostring Technologies, Inc., USA). After incubation at 65 °C for 19 hours, samples were processed on a Nanostring nCounter FLEX platform. Data analysis was performed using freely-available nSolver™ analysis software (version 4.0; NanoString Technologies, Inc.). The mRNA profiling data was normalized based on individual digital molecular barcodes to the expression levels determined for six specific housekeeping genes. Background signals that were estimated from blank wells were subtracted from the raw values. P-value adjustments were performed using the Benjamini-Hochberg false discovery rate (FDR) estimation method. Differentially expressed genes with a fold-change ≤ 2 and ≥ 2 and FDR-adjusted *p*-values ≤ 0.05 were selected.

CODESET DETAILS

	Gene	Accession number	Position
1	CCND1	NM_053056.2	691-790
2	CD44	NM_001001392.1	430-529
3	COL17A1	NM_000494.3	5071-5170
4	COL18A1	NM_030582.3	5792-5891
5	COL4A1	NM_001845.4	781-880
6	COL4A2	NM_001846.2	1151-1250
7	COL4A5	NM_000495.4	1715-1814
8	COL4A6	NM_001847.2	1071-1170
9	COL7A1	NM_000094.2	391-490
10	CSPG4	NM_001897.4	7643-7742
11	CTNNB1	NM_001098210.1	1816-1915
12	DAG1	NM_001165928.2	966-1065
13	E2F1	NM_005225.1	936-1035
14	EGFR	NM_201282.1	1355-1454
15	FBLN1	NM_006487.2	966-1065
16	FBLN2	NM_001004019.1	3441-3540
17	FOXM1	NM_202002.1	1001-1100

18	GAPDH	NM_001256799.1	387-486
19	HPRT1	NM_000194.1	241-340
20	HSPG2	NM_005529.5	2716-2815
21	ITGA3	NM_002204.2	1117-1216
22	ITGA6	NM_000210.1	3066-3165
23	ITGB1	NM_033666.2	2001-2100
24	ITGB4	NM_001005731.1	4152-4251
25	KLF4	NM_004235.4	1981-2080
26	KRT14	NM_000526.4	524-623
27	KRT15	NM_002275.3	1481-1580
28	LAMA3	NM_000227.3	4261-4360
29	LAMA5	NM_005560.3	788-887
30	LAMB1	NM_002291.2	3121-3220
31	LAMB3	NM_000228.2	3351-3450
32	LAMC1	NM_002293.3	4916-5015
33	LAMC2	NM_005562.2	491-590
34	LEF1	NM_016269.3	1166-1265
35	LRIG1	NM_015541.2	4323-4422
36	MKI67	NM_002417.2	4021-4120
37	MMP14	NM_004995.2	1471-1570
38	NF2	NM_000268.3	1896-1995
39	NGFR	NM_002507.3	2731-2830
40	NID1	NM_002508.2	3546-3645
41	NID2	NM_007361.3	1996-2095
42	PGK1	NM_000291.2	1031-1130
43	PIEZO1	NM_001142864.1	446-545
44	ROCK1	NM_005406.1	2661-2760
45	RPLP0	NM_001002.3	251-350
46	SPARC	NM_003118.3	975-1074
47	SRF	NM_003131.2	4041-4140
48	TAFAZZIN	NR_024048.1	1246-1345
49	TFRC	NM_003234.1	1221-1320
50	TP63	NM_003722.4	1296-1395
51	TUBB	NM_178014.2	1956-2055
52	YAP1	NM_001130145.2	4424-4523
53	YWHAZ	NM_003406.2	2346-2445

REFERENCES

Bachy, S., Letourneur, F., & Rousselle, P. (2008). Syndecan-1 interaction with the LG4/5 domain in laminin-332 is essential for keratinocyte migration. *J Cell Physiol*, *214*(1), 238-249. https://doi.org/10.1002/jcp.21184

Christiano, A. M., Anton-Lamprecht, I., Amano, S., Ebschner, U., Burgeson, R. E., & Uitto, J. (1996). Compound heterozygosity for COL7A1 mutations in twins with dystrophic epidermolysis bullosa: a recessive paternal deletion/insertion mutation and a

dominant negative maternal glycine substitution result in a severe phenotype. *Am J Hum Genet*, *58*(4), 682-693.
Decline, F., & Rousselle, P. (2001). Keratinocyte migration requires alpha2beta1 integrin-mediated interaction with the laminin 5 gamma2 chain. *J Cell Sci*, *114*(Pt 4), 811-823.
Dos Santos, M., Michopoulou, A., André-Frei, V., Boulesteix, S., Guicher, C., Dayan, G., Whitelock, J., Damour, O., & Rousselle, P. (2016). Perlecan expression influences the keratin 15-positive cell population fate in the epidermis of aging skin. *Aging (Albany NY)*, *8*(4), 751-768. https://doi.org/10.18632/aging.100928
Lunstrum, G. P., Sakai, L. Y., Keene, D. R., Morris, N. P., & Burgeson, R. E. (1986). Large complex globular domains of type VII procollagen contribute to the structure of anchoring fibrils. *J Biol Chem*, *261*(19), 9042-9048.
Paulsson, M., Deutzmann, R., Dziadek, M., Nowack, H., Timpl, R., Weber, S., & Engel, J. (1986). Purification and structural characterization of intact and fragmented nidogen obtained from a tumor basement membrane. *Eur J Biochem*, *156*(3), 467-478. https://doi.org/10.1111/j.1432-1033.1986.tb09605.x
Rousselle, P., Lunstrum, G. P., Keene, D. R., & Burgeson, R. E. (1991). Kalinin: an epithelium-specific basement membrane adhesion molecule that is a component of anchoring filaments. *J Cell Biol*, *114*(3), 567-576. https://doi.org/10.1083/jcb.114.3.567
Sakai, L. Y., Keene, D. R., Morris, N. P., & Burgeson, R. E. (1986). Type VII collagen is a major structural component of anchoring fibrils. *J Cell Biol*, *103*(4), 1577-1586. https://doi.org/10.1083/jcb.103.4.1577
