# Supplementary material for: Dermal stiffness governs the topography of the epidermis and the underlying basement membrane in young and old human skin
Source: Aging Cell. 2024 Mar 12;23(4):e14096. doi: 10.1111/acel.14096 (PMC11019137; doi:10.1111/acel.14096)
Supplement: Supplementary file 1 — Data S1: [file ACEL-23-e14096-s001.zip › 3_Supporting information revised.pdf]

Supporting information 1

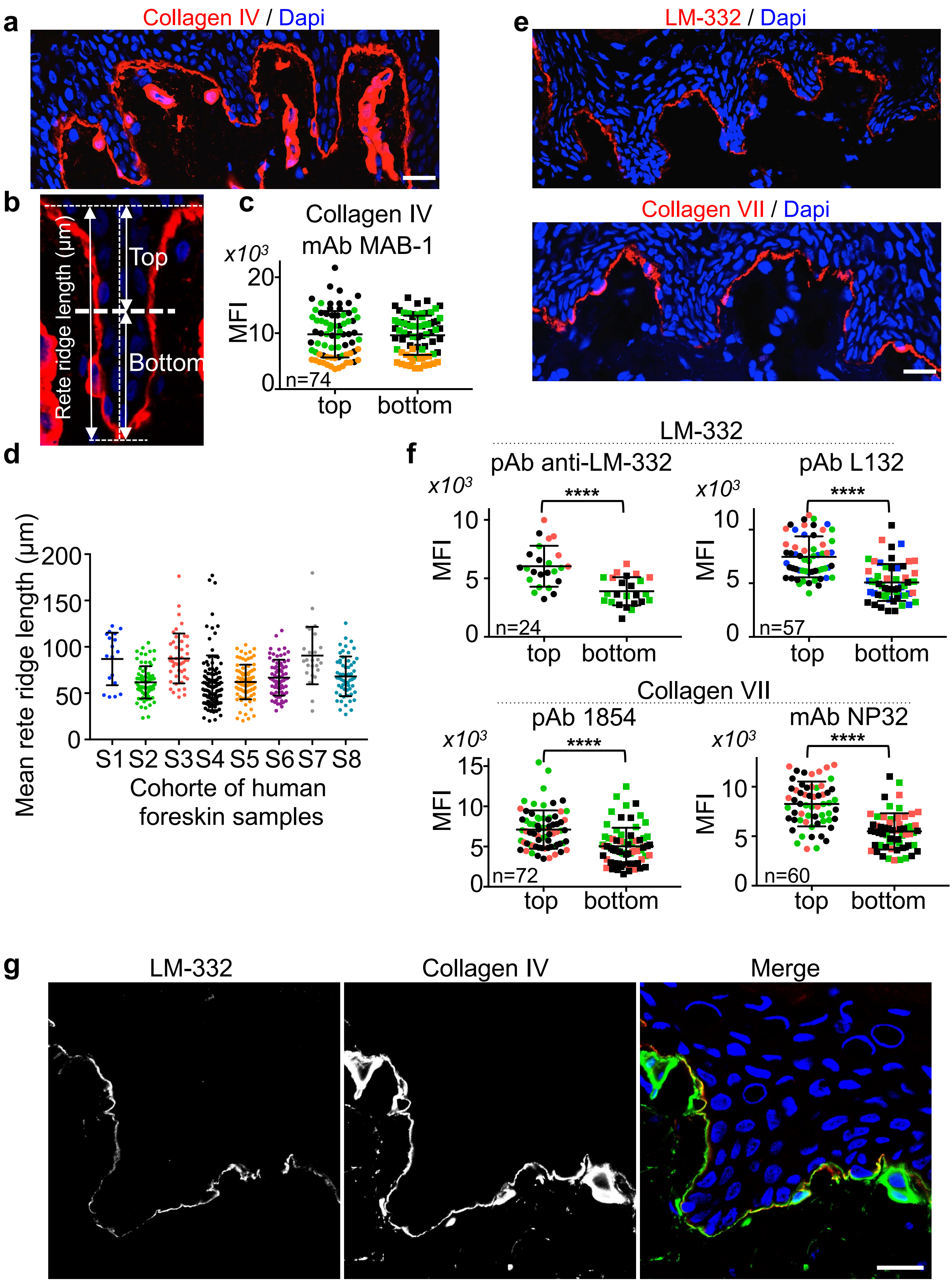

Figure S1. Analysis of basement membrane components in the rete ridges of human foreskin samples. (a) Immunofluorescence staining of collagen IV on frozen sections of human foreskin. The primary antibody was detected with CY3-labelled antibodies and nuclei were stained with DAPI. Composite images of CY3-labelled staining (red) and DAPI staining (blue) is shown. Bars, 20 µm.(b) Diagram explaining the measurements of rete ridge length and mean fluorescence intensity (MFI) on the top and bottom of the rete ridges (c) MFI of collagen IV at the top and bottom of the rete-ridges. Each point cloud of a given color represents the population of rete ridges analyzed in the color-assigned biopsy as shown in (d) in 3 independent experiments. (d) Length of epidermal rete ridges in human foreskins. Mean values are 86.89, 61.71, 87.44, 61.30, 62.05, 66.71, 90.56 and 68.14 µm from sample S1 to S8, respectively. Indicated are mean values ± SD (n = 18, 75, 45, 106, 84, 72, 25, 58 from S1 to S8). (e) Immunofluorescence staining of LM-332 and collagen VII on frozen sections of human foreskin. The primary antibody was detected with CY3-labelled antibodies and nuclei were stained with DAPI. Composite images of CY3-labelled staining (red) and DAPI staining (blue) are shown. Bars, 20 µm.(f) MFI of LM-332 and collagen VII at the top and bottom of the rete-ridges. Each point cloud of a given color represents the population of rete ridges analyzed in the color-assigned biopsy as shown in (d) in 3 independent experiments. (c, f) In each case, biopsies from 3 different donors were analyzed (n are indicated). Shown are means ± SD with ****p < 0.0001, determined with a Student's t-test in relation to MFI at the top of the rete ridge. (g) Double immunostaining of LM-332 and collagen IV detected with CY3 and FITC-labelled antibodies respectively, showing both their colocalized and distinct labeling patterns along the DEJ on frozen sections of human foreskin. Black and white single and merged staining with DAPI (blue) are shown as indicated. Bar, 20 µm.

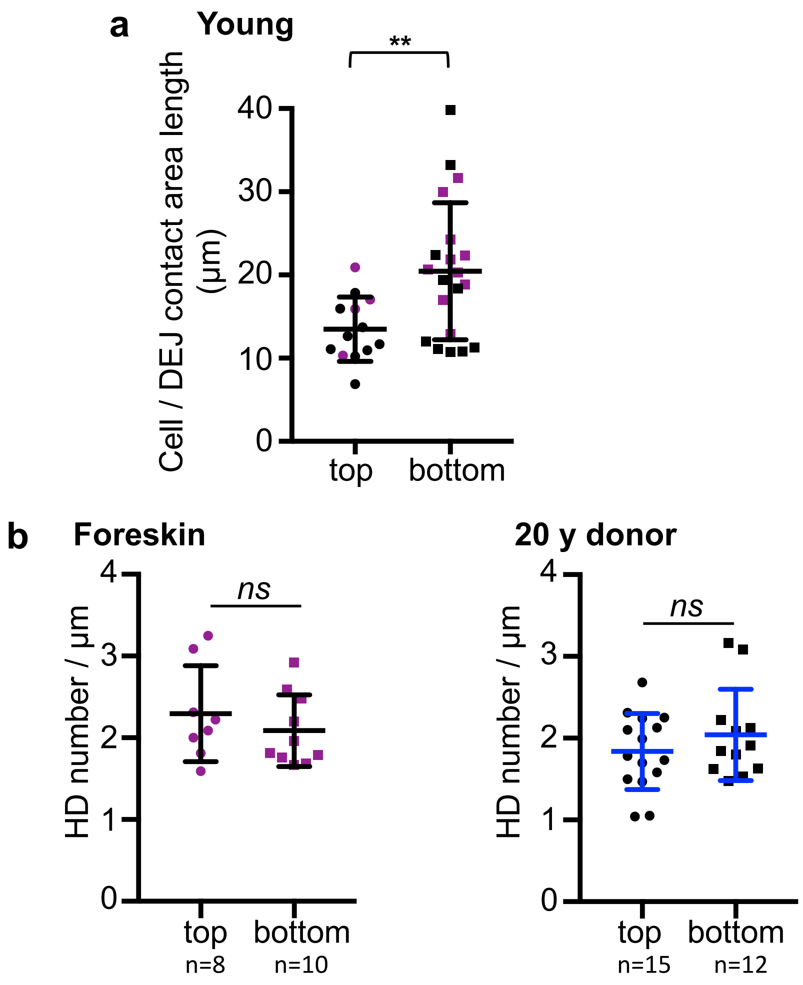

Figure S2. Contact area between cell and DEJ at the upper and lower ends of epidermal rete ridges. (a) Length of membrane contact between basal keratinocytes and the BM at the upper and lower ends of the rete ridges of young skin. (b, c) Number of HDs / basal cell membrane µm in foreskin and 20-year-old biopsy. The number of keratinocytes in which the contact zone was measured and HDs counted is indicated in each scatter plot (n). The purple and black symbols represent data from a foreskin sample and a 20-year-old female donor biopsy, respectively. **p < 0.001 vs. control, Student’s t-test. ns, not significant.

Supporting information 3

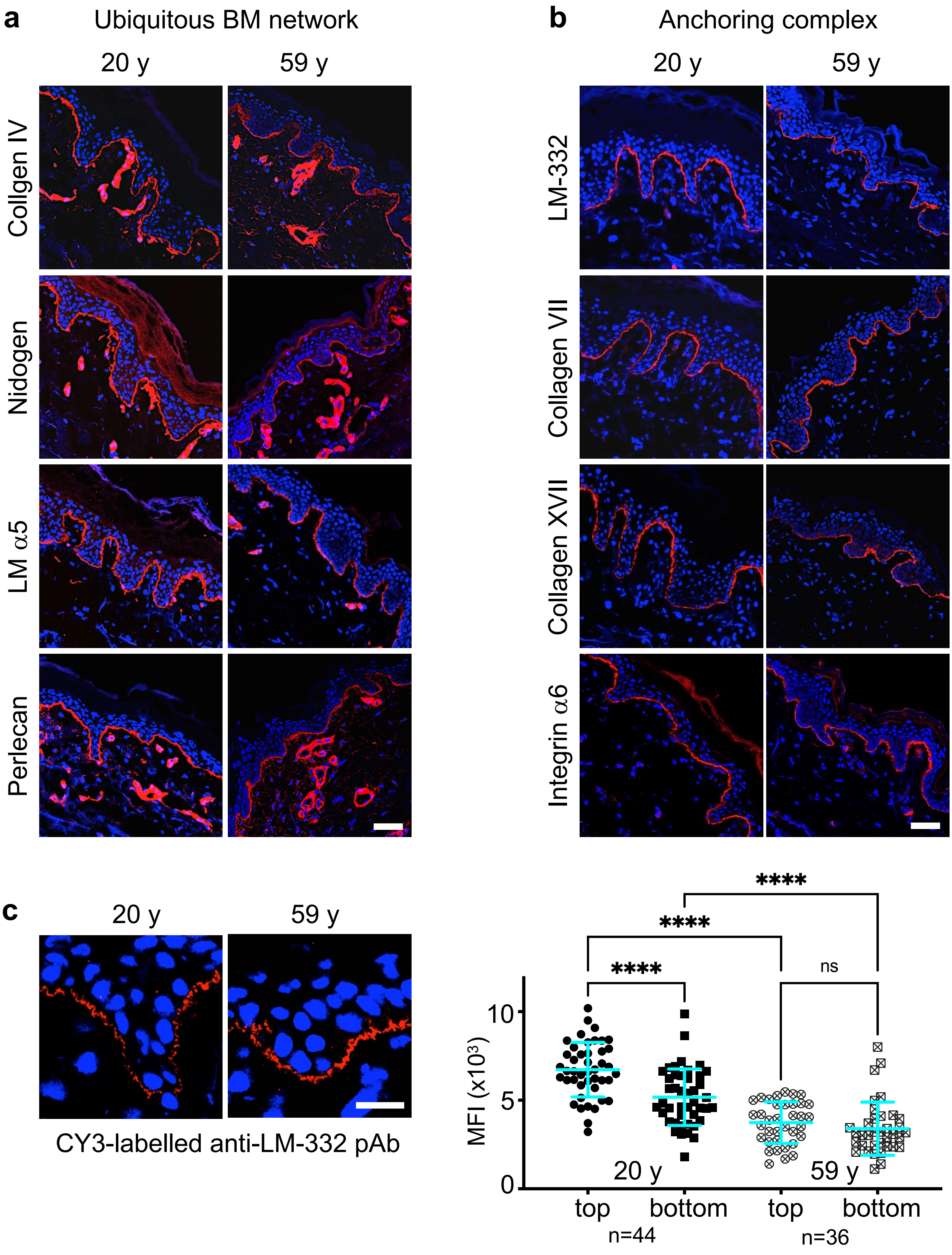

d

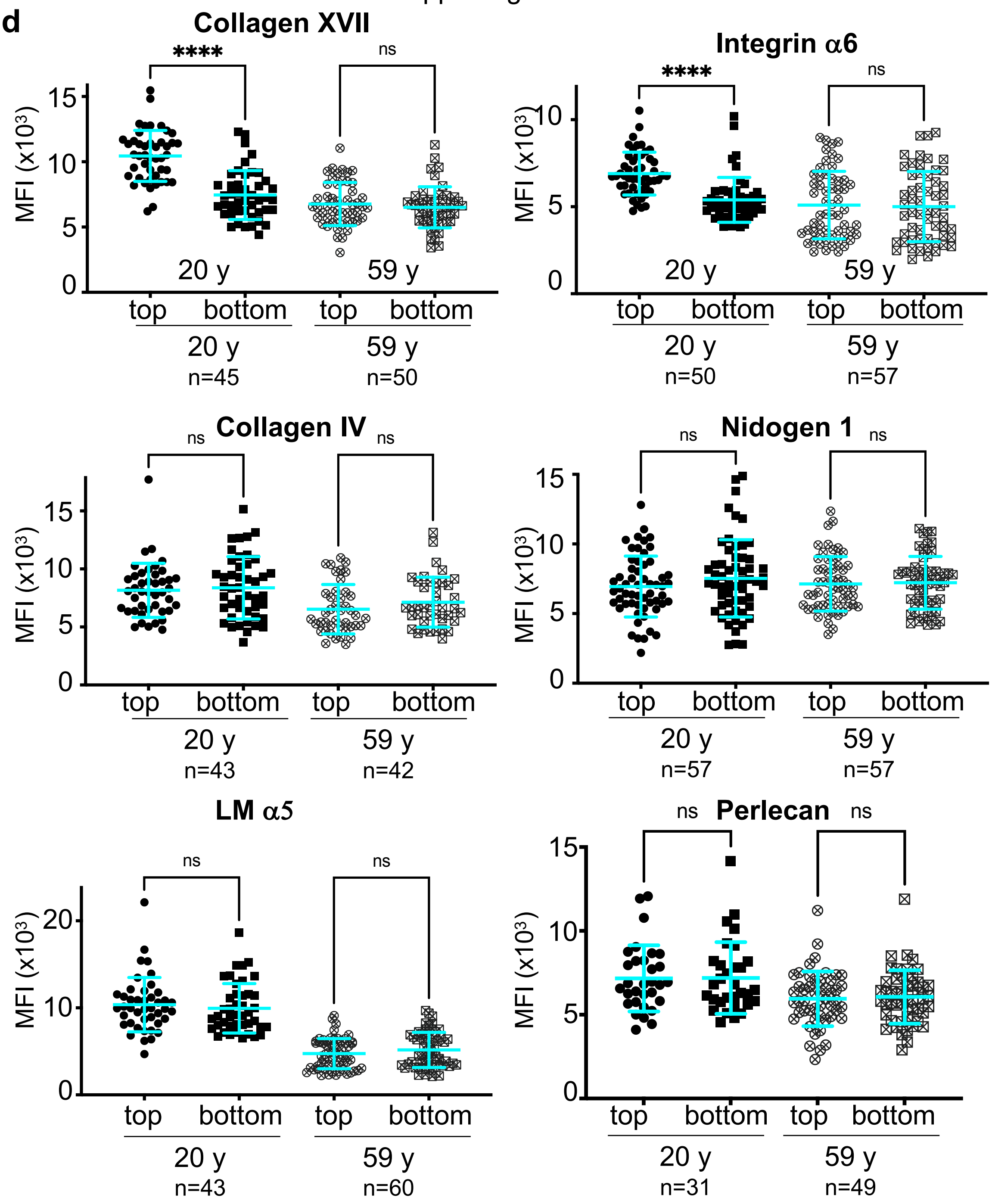
Collagen XVII
****
ns
MFI (x10³)
20 y
59 y
top bottom top bottom
20 y
n=45
59 y
n=50
Integrin α6
****
ns
n=50
n=57
Collagen IV
ns
ns
n=43
n=42
Nidogen 1
ns
ns
n=57
n=57
LM α5
ns
ns
n=43
n=60
Perlecan
ns
ns
n=31
n=49

e

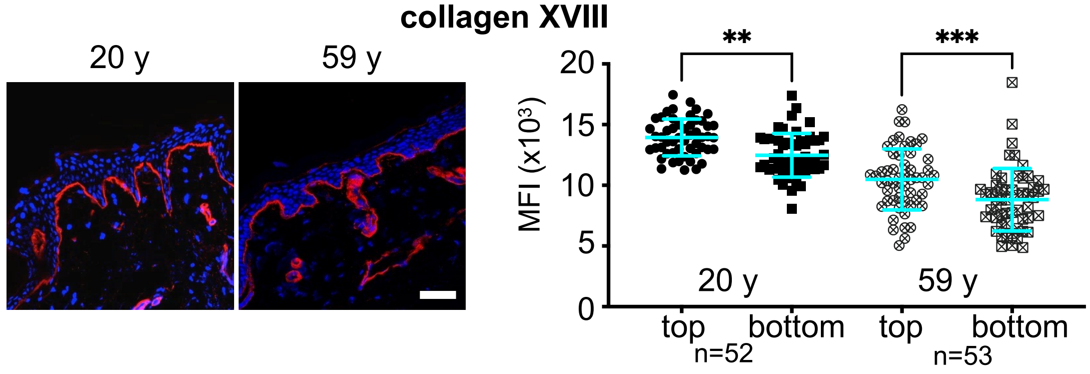
collagen XVIII
20 y
59 y
**
***
MFI (x10³)
top bottom top bottom
n=52
n=53

Figure S3. Expression of DEJ components along epidermal rete ridges during skin ageing. (a, b) Immunofluorescence staining of collagen IV, nidogen, LM-511 and perlecan (a) and LM-332, collagens VII and XVII and integrin α6 (b) on frozen sections of abdominal skin from donors aged 20 and 59 years. Each specific primary antibody was detected by CY3-labelled antibodies and the nuclei were stained with DAPI. Images of CY3-labelled staining (red) and DAPI staining (blue) are combined. Bar, 50 µm. (c) Left, immunofluorescence staining of the young and aged samples described above using a CY3-labelled anti LM-332 pAb (red) and DAPI (blue). Bar, 20 µm. Right, MFI of the CY3-labelled pAb at the top and bottom of the rete-ridges. (d) MFI of each indicated DEJ component at the top and bottom of the rete ridges. (e) Left, immunofluorescence staining of collagen XVIII on frozen sections of abdominal skin from donors aged 20 and 59 years. Images show merge of CY3-labelled staining (red) and DAPI (blue). Bar, 20 µm. Right, MFI of collagen XVIII at the top and bottom of the rete ridges in the indicated skin biopsies. (c, d, e) Each point cloud represents the population of rete ridges analysed in the skin biopsies of 20 and 59 year old donors from 3 independent experiments. Shown are means ± SD with ****$p < 0.0001$, ***$p < 0.001$, **$p < 0.01$, determined with an Anova test (n are indicated). ns, not significant.

Supporting information 4

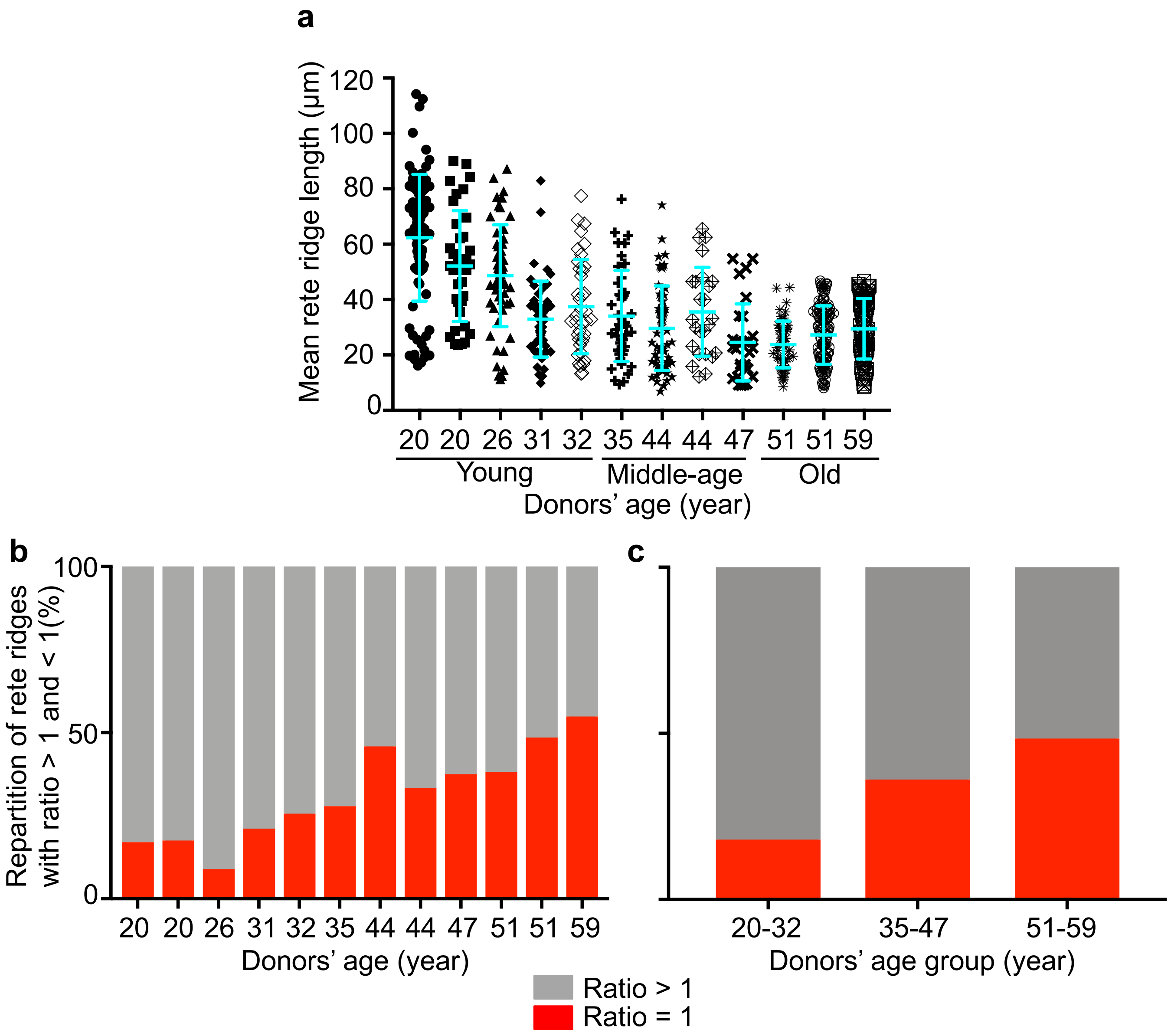

Figure S4. Rete ridges with a low LM-332 uniform expression increase with age. (a) Length of epidermal rete ridges in a cohort of human abdominal samples from female donors. n values are 87, 36, 56, 52, 39, 43, 61, 25, 29, 53, 94 and 78 in order of age indicated in the graph. Indicated are mean values ± SD. (b) Proportion of epidermal rete ridges lacking LM-332 gradient expression out of the total number of epidermal rete ridges analysed (red columns) per biopsy. n-values are 41, 40, 56, 52, 39, 43, 61, 27, 40, 50, 35 and 51 in order of age in the graph. (c) Proportion of epidermal rete ridges lacking the LM-332 top/bottom gradient (red columns) divided into three age groups as in Figure 4b.

Supporting information 5

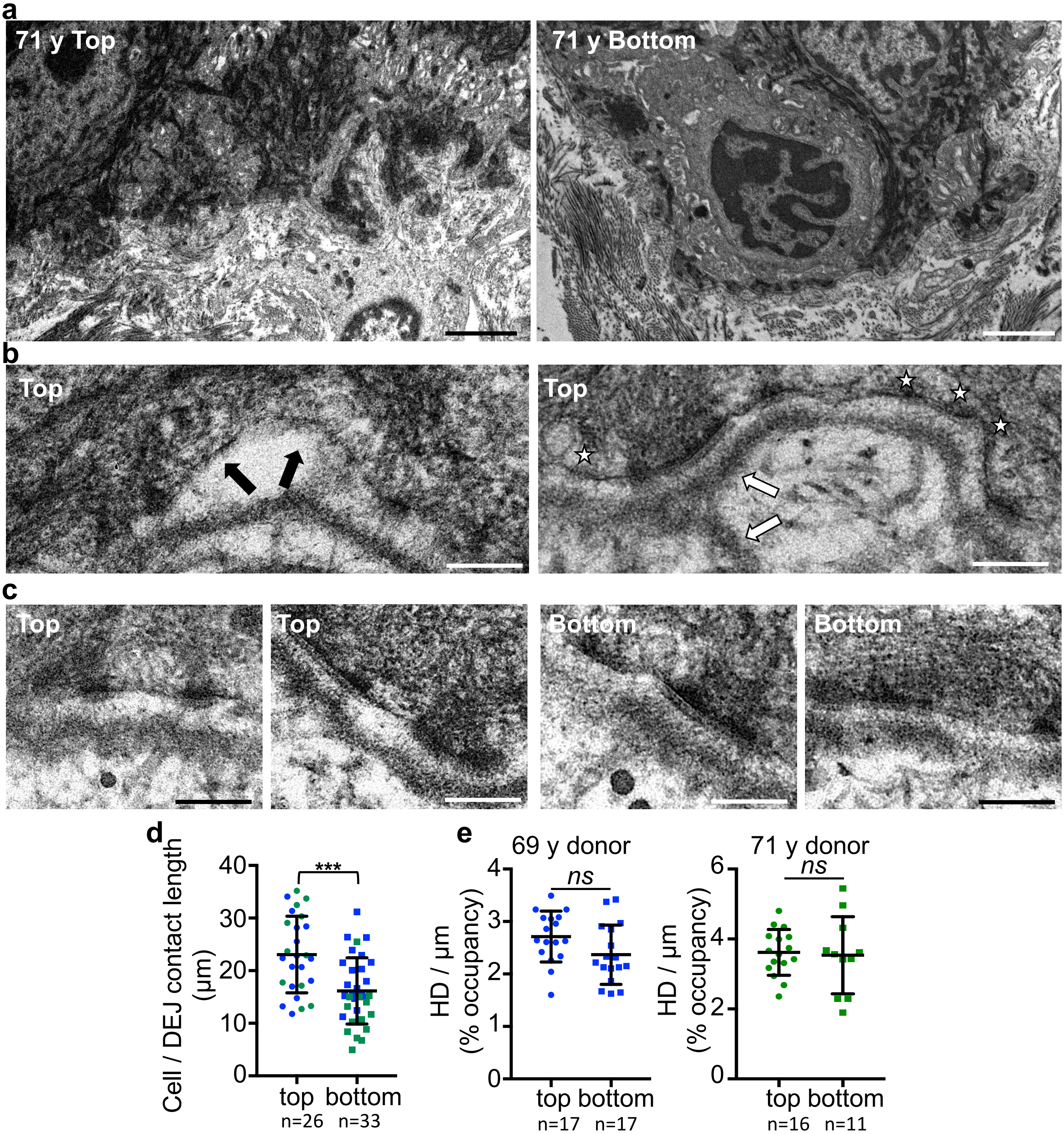

Figure S5. TEM Analysis of DEJ in aged skin. (a, b, c) TEM Image of DEJ (a, b) and HDs (c) at the top and bottom of the rete ridge in the skin of a 71-year-old female donor as indicated. Scale bars 2 μm (a), 200 nm (b, c). (b) TEM Images of the DEJ show the main age-related defects such as detachment of keratinocytes (black arrow), doubling of the lamina densa (white arrows) and the presence of nascent HDs (asterisks). (d) Length of membrane contact between basal keratinocytes and BM at the top and bottom of the rete-ridges. (e) Number of HDs / μm basal cell membrane in skin from 69- and 71-year-old donors. (d, e) The contact zone was measured (d) or the number of keratinocytes in which the HDs were numbered (e) is indicated. Blue and green symbols represent data from a 69- and a 71-year-old donor, respectively, as indicated. ****p < 0.0001 vs. control, Student's t-test. ns, not significant.

Supporting information 6

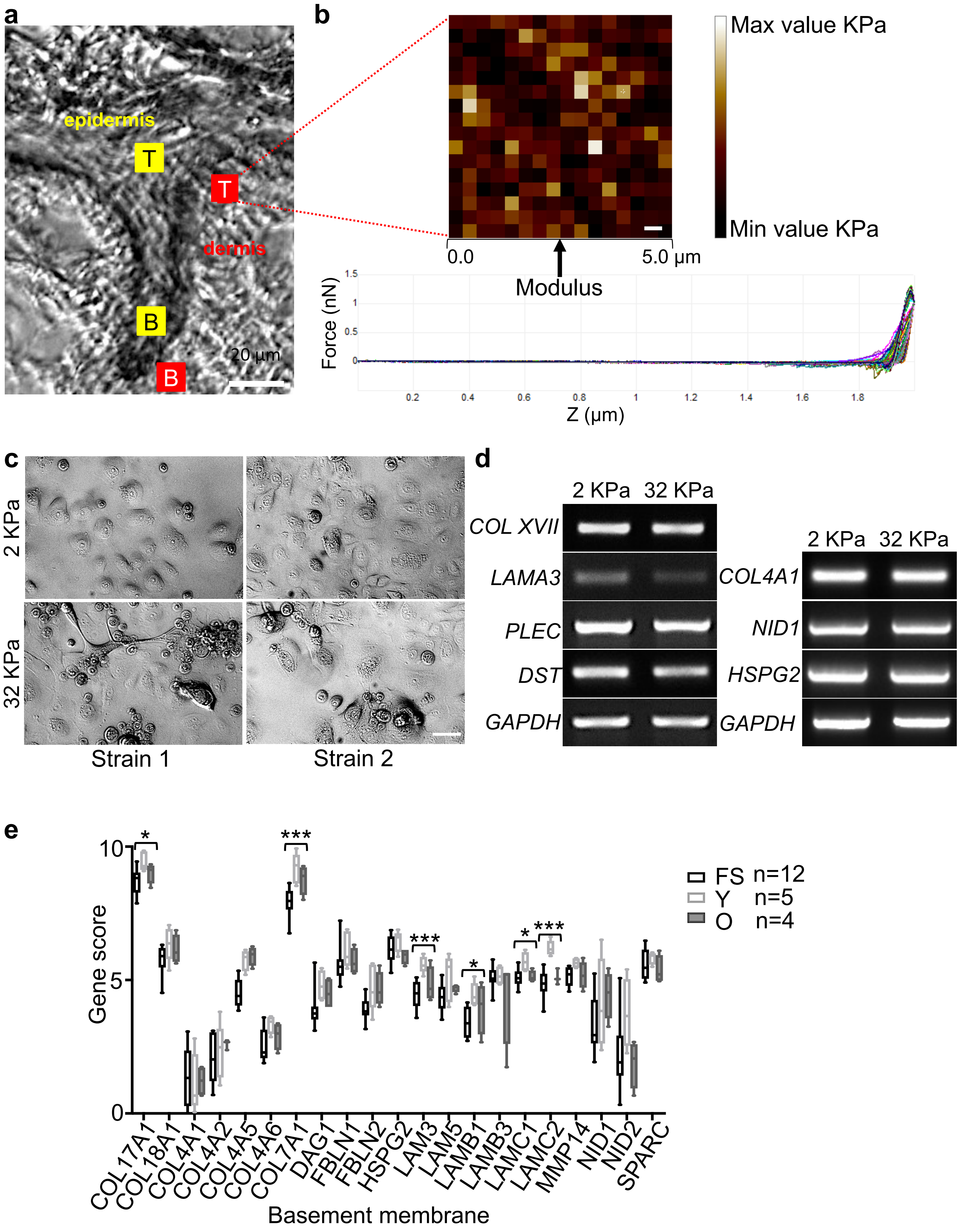

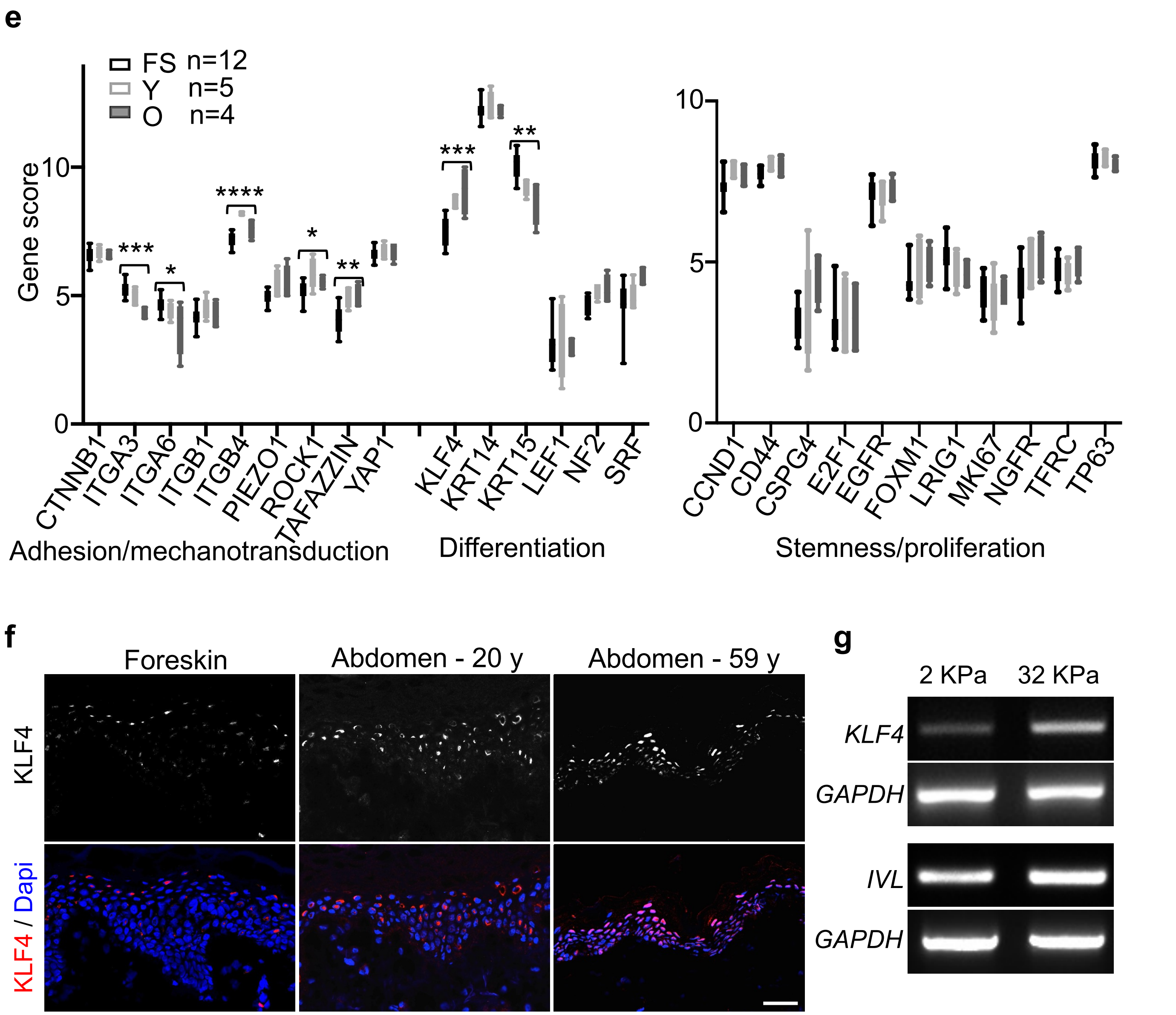

Figure S6. Stiffness of the matrix determines the fate of hemidesmosomes. (a) Atomic force microscopy (AFM) study of human skin. Sagittal tissue sections (10 µm) were prepared from full thickness skin biopsies and used for AFM measurements. The AFM cantilever with a spherical probe tip of 140 nm diameter was navigated either in the epidermis (yellow) or in the dermal matrix (red) on the top (T) and bottom (B) of the epidermal rete ridges using light microscopy, bar 20 µm. (b) Lower graph, force-displacement curves (F-z) were generated for each point by plotting the deflection of the cantilever against the controlled deformation. E was calculated by fitting the contact part of the force-displacement curves (F-d) using a standard Hertz model. Upper graph, for all samples, 16 x 16 points were examined in a 5 x 5 µm area as shown in the color-coded maps, scale bar 300 nm. (c, d) C-DIC phase contrast images of two strains of primary human keratinocytes (strain 1: foreskin, strain 2: abdomen at age 32) cultured for 48 h on surfaces with stiffness of 2 KPa and 32 KPa, as indicated. Bar, 50 µm and (d) *COL17A1*, *LAMA3*, *PLEC, DST, COL4A1, NID1 , HSPG2 and GAPDH* mRNA expression from keratinocytes submitted to each condition obtained by RT-PCR.

(e) NanoString nCounter® transcriptomic differential analysis of genes grouped into categories in keratinocytes from foreskin (FS) epidermal rete ridges compared to abdominal skin from young (Y) and old (O) donors. Shown are means ± SD with ****$p < 0.0001$, ***$p < 0.001$, **$p < 0.01$, *$p < 0.1$ determined with an Anova test (n are indicated). No indication means not significant. (f) Immunofluorescence staining of KLF4 on human frozen sections from human biopsies of the indicated origin. KLF4 antibody was detected with CY3-labelled antibody and nuclei were stained with DAPI. Images of CY3-labelled staining (red) and DAPI staining (blue) are combined. Bar, 40 µm. (g) (F) *KLF4, GAPDH and IVL, GAPDH* mRNA expression obtained by RT-PCR from primary human keratinocytes cultured for 48 h on surfaces with stiffness of 2 KPa and 32 Kpa as described in (c) The PCR for *IVL* was performed in the same series of experiments with the genes *COL4A1*, *NID1* and *HSPG2* (see Fig. S6d), which explains that the GAPDH bands are the same.
